# Generation and Reactivity of 1-Imidocarbenium Cations in the Friedel–Crafts-type Reaction

**DOI:** 10.1021/acsomega.2c03930

**Published:** 2022-08-15

**Authors:** Jakub Adamek, Roman Mazurkiewicz, Anna Węgrzyk-Schlieter

**Affiliations:** † Department of Organic Chemistry, Bioorganic Chemistry and Biotechnology, Silesian University of Technology, Bolesława Krzywoustego 4, 44-100 Gliwice, Poland; ‡ Biotechnology Center, Silesian University of Technology, Bolesława Krzywoustego 8, 44-100 Gliwice, Poland

## Abstract

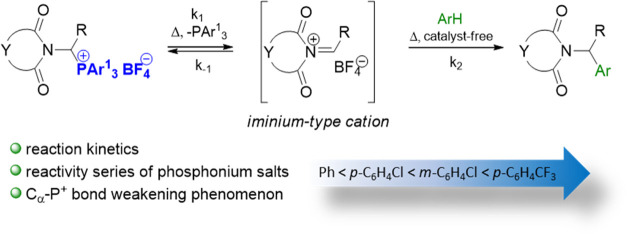

Herein, we discuss the formation and reactivity of 1-imidocarbenium cations in Friedel–Crafts-type reaction between 1-imidoalkylphosphonium salts and arenes. The observed weakening of C_α_–P^+^ bond is described qualitatively and quantitatively. The determination of rate constants and activation energies of C_α_–P^+^ bond cleavage enabled systematic reactivity investigations of a series of phosphonium salts with different structures. Finally, the application scope for the imidoalkylation of aromatic hydrocarbons was explored. The results confirm that the generated 1-imidocarbenium cations are reactive enough to alkylate strongly activated, less-activated, or even inactivated aromatic compounds.

## Introduction

**Scheme 1:**
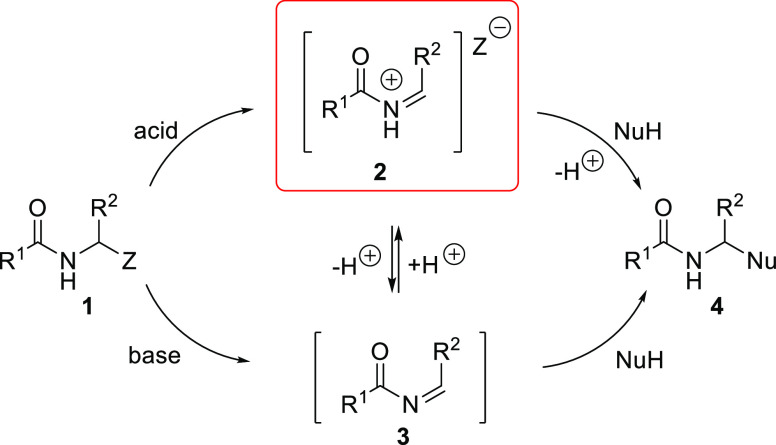
Generation of *N*-Acyliminium Cations **2** and *N*-Acylimines **3** during α-Amidoalkylation Reactions

α-Amidoalkylation reactions have recently garnered significant attention owing to their efficiency for (N)C–C and (N)C–heteroatom bond formations. Importantly, these reactions can be used to form β-aminocarbonyl substructures or to construct new carbo- or heterocyclic motifs, which have applications in pharmaceutical chemistry and natural products synthesis.^1−9^*N*-Acylimines **3** and much more reactive *N*-acyliminium cations **2** are active intermediates in α-amidoalkylation reactions; these compounds are usually generated in situ from a secondary amide precursor **1** under basic or acidic conditions, respectively (Scheme 1).^3−5^

**Scheme 2:**
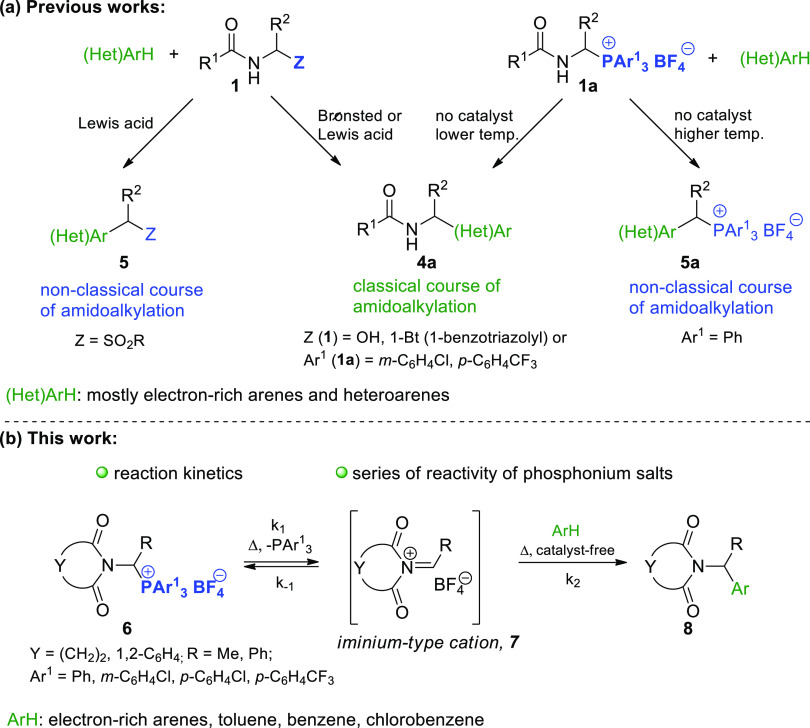
Modern Strategies for the α-Amidoalkylation of Aromatic Compounds

*N*-Acyliminium cations **2** are important and highly reactive intermediates with a broad application scope in organic synthesis recognized over the last decade^1−4,8,9^ and earlier.^5−7^ However, *N*-acyliminium cations exhibit insufficient reactivity toward various nucleophiles that are relatively inert, e.g., aromatic systems. This low reactivity limits the scope of α-amidoalkylation reactions involving arenes (i.e., Tscherniac–Einhorn-type amidoalkylation or, more generally, Friedel–Crafts-type alkylation) to aromatic compounds with strong electron-donating substituents (e.g., alkoxy-, polyalkoxy-, and aminoarene groups) and some active heterocycles (e.g., indoles). Furthermore, a strong acid catalyst, often in stoichiometric amounts, must be added to carry out the reaction. This requirement hinders and complicates the workup procedures, and it may alter the direction of the reaction, leading to the formation of unexpected products. Scheme 2a presents the classical and nonclassical α-amidoalkylation reaction pathways, respectively.^10−12^ The aforementioned limitations are particularly important for intramolecular α-amidoalkylations that afford new carbo- or heterocycles.

Our group previously demonstrated that the challenges associated with (i) the insufficient reactivity of *N*-acyliminium cations **2** toward generally inert nucleophiles and (ii) the resulting unexpected amidoalkylation reaction pathways can be addressed to a significant extent by replacing *N*-acyliminium cations **2** with much more electrophilic 1-imidocarbenium cations **7**.^13^ Herein, for the first time, we fully describe qualitatively and quantitatively the generation of such cations from precursors **6** under noncatalytic conditions. The kinetics governing C_α_–P^+^ bond cleavage (which generates the corresponding cation **7**) were studied by introducing highly reactive arenes (e.g., anisole, 1,3-dimethoxybenzene, 1,3,5-trimethoxybenzene) to trap the cations of interest. The structure–reactivity correlations were investigated for selected phosphonium precursors of the iminium-type cations (Scheme 2b).

## Results and Discussion

Several years ago, we developed a method for the efficient synthesis of previously unexplored 1-(*N*-acylamino)alkylphosphonium salts (**1a**; Z = PPh_3_^+^ X^–^, X = Br, I, BF_3_) and demonstrated their amidoalkylating capabilities.^14^ The key feature of these salts, which distinguishes them from other amidoalkylating agents **1**, is the permanent positive charge on the phosphonium leaving group; this positive charge eliminates the need for an acid catalyst when generating *N*-acyliminium cations **2**.

**Scheme 3:**
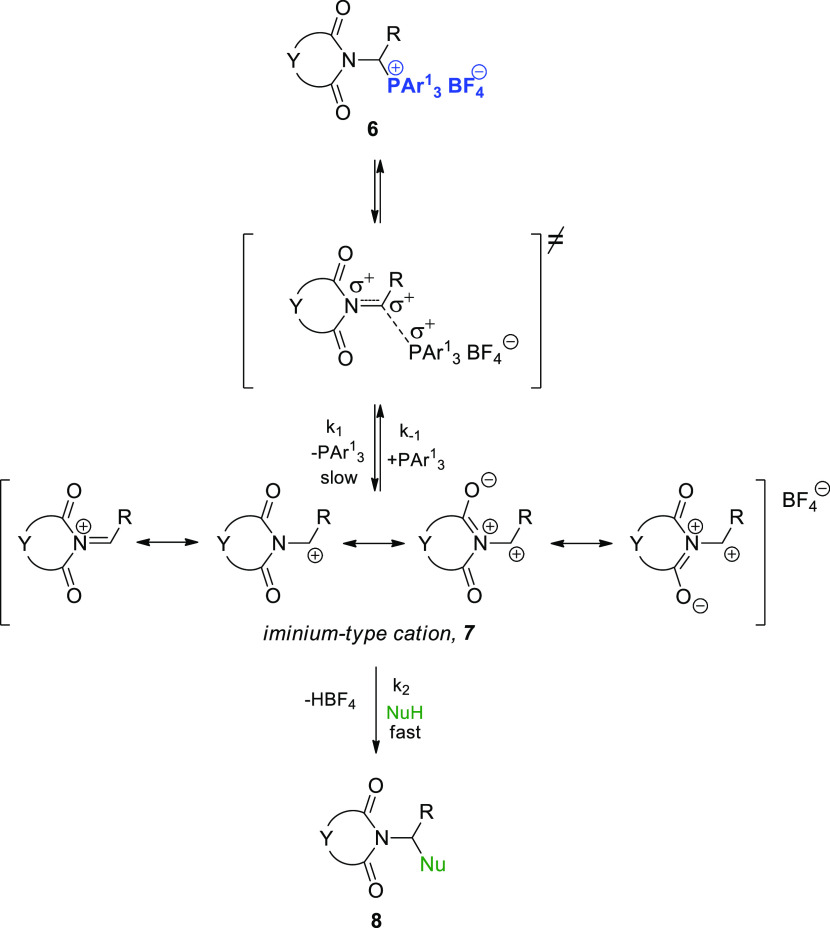
Generation of Iminium-Type Cations **7** from 1-Imidoalkylphosphonium salts **6**

We also described the synthesis of structurally similar 1-imidoalkylphosphonium salts **6**.^13^ It is reasonable to assume that 1-imidoalkylcarbenium cations **7** generated from these salts should exhibit greater electrophilic reactivity relative to *N*-acyliminium cations **2** owing to the strong electron-withdrawing effect of the two carbonyl groups adjacent to the nitrogen atom (Scheme 3). Moreover, there is no deprotonation reaction, which in the case of 1-(*N*-acylamino)alkylphosphonium salts **1a** (Scheme 3 vs 1) additionally reduces their reactivity. We hypothesized that replacing *N*-acyliminium cations with 1-imidocarbenium cations would enable the formation of a carbon–carbon bond (i.e., (N)C–C) with C-nucleophiles with low reactivity (e.g., less-activated, inactivated, or even deactivated arenes).

The kinetics governing the imidoalkylation of aromatic hydrocarbons with 1-imidoalkylphosphonium salts **6** were systematically evaluated over the temperature range 60–160 °C using solutions containing the phosphonium salt and an excess of arenes, with or without additional cosolvent (Table 1). The reaction rates were determined by monitoring the disappearance of 1-imidoalkylphosphonium salts by ^1^H-NMR spectroscopy. The spectroscopic characterization of the relevant substrates and products is described in our group’s previous work.^13^

**Table 1:**
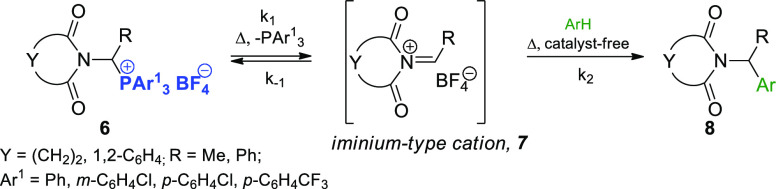
Kinetics of the Reactions between 1-Imidoalkylphosphonium Salts **6** and Various Aromatic Compound

	1-imidoalkylphosponium salts^a^							
entry	no	Y	R	Ar^1^	ArH^b^	cosolvent^c^	temp. [°C]	*k*_1_ × 10^4^ [s^–1^]
1	**6a**	*o*-C_6_H_4_	Me	*m*-Cl-C_6_H_4_	C_6_H_5_OMe	PhNO_2_	100	0.80
2	**6a**	*o*-C_6_H_4_	Me	*m*-Cl-C_6_H_4_	1,3-C_6_H_4_(OMe)_2_	PhNO_2_	100	1.08
3	**6a**	*o*-C_6_H_4_	Me	*m*-Cl-C_6_H_4_	1,3,5-C_6_H_3_(OMe)_3_	PhNO_2_	100	1.19
4	**6a**	*o*-C_6_H_4_	Me	*m*-Cl-C_6_H_4_	C_6_H_5_OMe	PhNO_2_	120	7.50
5	**6a**	*o*-C_6_H_4_	Me	*m*-Cl-C_6_H_4_	1,3-C_6_H_4_(OMe)_2_	PhNO_2_	120	6.86
6	**6a**	*o*-C_6_H_4_	Me	*m*-Cl-C_6_H_4_	1,3,5-C_6_H_3_(OMe)_3_	PhNO_2_	120	10.3
7	**6a**	*o*-C_6_H_4_	Me	*m*-Cl-C_6_H_4_	C_6_H_5_OMe + 1,3-C_6_H_4_(OMe)_2_^d^	PhNO_2_	100	1.09^e^
8	**6a**	*o*-C_6_H_4_	Me	*m*-Cl-C_6_H_4_	1,3-C_6_H_4_(OMe)_2_ + 1,3,5-C_6_H_3_(OMe)_3_^d^	PhNO_2_	100	1.31^f^
9	**6b**	*o*-C_6_H_4_	Me	*p*-CF_3_-C_6_H_4_	1,3-C_6_H_4_(OMe)_2_	PhNO_2_	100	6.0
10	**6b**	*o*-C_6_H_4_	Me	*p*-CF_3_-C_6_H_4_	1,3-C_6_H_4_(OMe)_2_	C_6_H_5_Me	100	18.7
11	**6b**	*o*-C_6_H_4_	Me	*p*-CF_3_-C_6_H_4_	1,3-C_6_H_4_(OMe)_2_	none^g^	100	16.1
12	**6c**	*o*-C_6_H_4_	Me	*p*-Cl-C_6_H_4_	1,3-C_6_H_4_(OMe)_2_	PhNO_2_	120	1.37
13	**6d**	*o*-C_6_H_4_	Me	Ph	1,3-C_6_H_4_(OMe)_2_	PhNO_2_	160	1.68
14	**6e**	*o*-C_6_H_4_	Ph	*m*-Cl-C_6_H_4_	1,3-C_6_H_4_(OMe)_2_	PhNO_2_	60	1.24
15	**6f**	(CH_2_)_2_	Me	*m*-Cl-C_6_H_4_	1,3-C_6_H_4_(OMe)_2_	PhNO_2_	140	1.81

a 0.08 mmol.

b 1.19 mmol (129 μL anisole, 156 μL 1,3-dimethoxybenzene, or 200 mg 1,3,5-trimethoxybenzene).

c 400 μL.

d Molar ratio of 1:1 (0.6:0.6 mmol).

e No traces of the α-imidoalkylation product of anisole were detected.

f Mixture of α-imidoalkylation products (1,3-C_6_H_4_(OMe)_2_ and 1,3,5-C_6_H_4_(OMe)_3_ in a molar ratio of 1:2).

g An additional 400 μL 1,3-C_6_H_4_(OMe)_2_ was used instead of a cosolvent.

The results of the first series of kinetic experiments (Table 1, entries 1–3) showed that 1-(*N*-phthalimido)ethyltris(*m*-chlorophenyl)phosphonium tetrafluoroborate **6a** reacted with anisole, 1,3-dimethoxybenzene, and 1,3,5-trimethoxybenzene at 100 °C via first-order kinetics with similar rate constants (0.80 × 10^–4^, 1.08 × 10^–4^, and 1.19 × 10^–4^ s^–1^, respectively). The same reactions carried out at 120 °C also gave relatively similar rate constants (7.50 × 10^–4^, 6.86 × 10^–4^, and 1.03 × 10^–3^ s^–1^, respectively; Table 1, entries 4–6).

The most striking result was observed for the mixture of 1,3-dimethoxybenzene and anisole in a molar ratio of 1:1 at 100 °C (Table 1, entry 7). In this experiment, the rate of disappearance of phosphonium salts **6a** was almost exactly the same as in the reaction using 1,3-dimethoxybenzene alone (1.09 × 10^–4^ and 1.08 × 10^–4^ s^–1^, respectively). The only reaction products were those generated following α-imidoalkylation of the 2- and 4-positions of 1,3-dimethexybenzene (in a molar ratio of 1:5.7). No evidence of anisole α-imidoalkylation was detected in this experiment. These results confirm that the reactivity of 1,3-dimethoxybenzene in the studied reaction is much higher than that of anisole. Similar conclusions were drawn from studies of other electrophilic substitutions of 1,3-dimethoxybenzene and anisole, e.g., the relative rate constants associated with the bromination of these compounds in acetic acid at 25 °C were estimated to be higher than 1:8·10^3^.^15^ An analogous experiment using an equimolar mixture of 1,3-dimethoxybenzene and 1,3,5-trimethoxbenzene (Table 1, entry 8) afforded a mixture of the expected corresponding α-imidoalkylation products in a molar ratio of 1:2, respectively (for 1,3-dimethoxybenzene, the sum of two formed isomers in a molar ratio of 1:6.5 was taken into account).

1-(*N*-Phthalimido)ethyltris(*m*-chlorophenyl)phosphonium tetrafluoroborate **6a** showed similar rates of disappearance during reactions with anisole, 1,3-dimethoxybenzene, and 1,3,6-trimethoxybenzene, despite the much lower reactivity of anisole relative to 1,3-dimethoxybenzene, which had noticeably lower reactivity than 1,3,5-trimethoxybenzene. This behavior can be explained by assuming that the first step of the reaction involved a slow, reversible cleavage of the C_α_–P^+^ bond in the phosphonium salt to yield 1-imidoalkylcarbenium cation **7**. This step was followed by a rapid reaction between the highly reactive cation and the most active aromatic hydrocarbon (Scheme 3). Considering the steady-state approximation, this reaction rate can be expressed using eq 1.
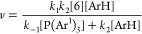
1

If *k*_–1_[P(Ar^1^)_3_] is negligible compared with *k*_2_[ArH], the higher reactivity and much higher concentration of ArH relative to P(Ar^1^)_3_ means that eq 1 can be simplified to eq 2, which is consistent with the first-order reaction kinetics of the S_N_1 mechanism.
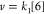
2

According to eq 2, the reaction rate does not depend on the type of aromatic hydrocarbon, assuming that *k*_–1_[P(Ar^1^)_3_] ≪ *k*_2_[ArH]. Moreover, the measured rate constants *k*_1_ (Table 1) correspond to the generation of 1-imidoalkylcarbenium cation **7** in the rate-determining step. These results indicate that the highly reactive 1,3-dimethoxybenzene or 1,3,5-trimethoxybenzene can be used to effectively trap 1-imidocarbenium cations **7**.

To evaluate the effect of the solvent polarity on the rate of generating 1-imidoalkylcarbenium cations, 1-(*N*-phthalimido)ethyltris(*p*-trifluormethylphenyl)phosphonium tetrafluoroborate **6b** (0.08 mmol) was heated at 100 °C with 1,3-dimethoxybenzene (1.19 mmol, 156 μL) either without a cosolvent or with nitrobenzene or toluene (400 μL) as a cosolvent (Table 1, entries 9–11). The phosphonium salt **6b** derived from tris(*p*-trifluoromethylphenyl)phosphine was used in these experiments owing to its relatively high solubility in low-polarity solvents. The measured reaction rates (6.00 × 10^–4^, 1.87 × 10^–3^, and 1.61 × 10^–3^ s^–1^, respectively, with nitrobenzene or toluene as a cosolvent or no cosolvent) indicated that a more polar solvent leads to a slower reaction (the dielectric constants of nitrobenzene, toluene, and 1,3-dimethoxybenzene are equal to 35.6, 2.4, and 5.4, respectively).^16^ This is a characteristic feature of S_N_1 reactions involving substrates with positively charged leaving groups. In such cases, the electric charge is more delocalized in the transition state than in the initial cation, and therefore, solvation in polar solvents more effectively reduces the energy of the ionic substrate more than that of the transition state (Scheme 3).

**Figure 1:**
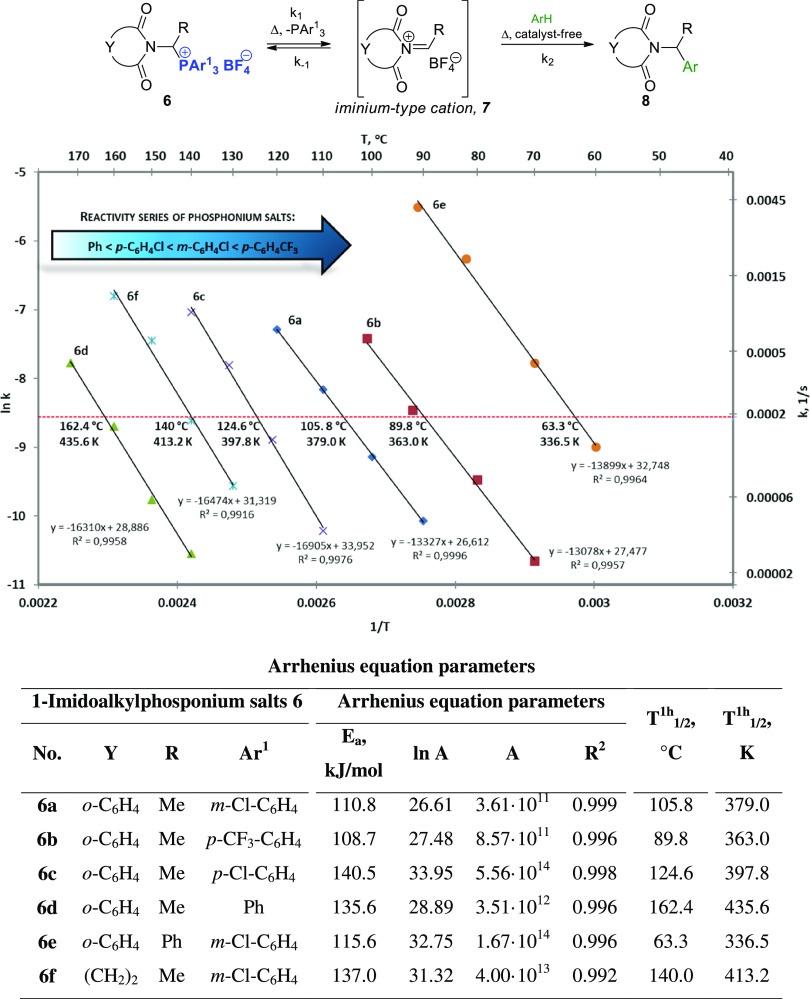
Arrhenius parameters (bottom) and plot of ln *k* as a function of 1/*T* (top) to describe the generation of 1-imidocarbenium cations **7** from the corresponding phosphonium salts **6a**–**f**.

To evaluate how the type of triarylphosphonium group and the 1-imidocarbenium cation structure impact the ease of cation generation, the rates of 1,3-dimethoxybenzene α-imidoalkylation were measured at various temperatures with nitrobenzene as the cosolvent. The results were used to calculate (i) the activation energies *E*_a_ corresponding to cation generation (based on the Arrhenius equation) and (ii) the temperature at which half of the initial phosphonium salts disappears after 1 h *T*_1/2_^1h^ (Figure 1). The latter denotes the temperature at which the α-imidocarbenium cation is generated from a phosphonium salt at a reasonable rate; this parameter can indicate that a particular salt is applicable under similar conditions as the α-imidoalkylation agent. The *T*_1/2_^1h^ can be calculated based on Arrhenius equation parameters using eq 3,
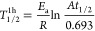
3where *A* is the Arrhenius frequency factor. The half-life *t*_1/2_ was set as 3600 s.

The *E*_a_ and *T*_1/2_^1*h*^ for the formation of 1-imidocarbenium cations derived from succinimide and phthalimide with methyl groups at the α-position were 137.0 and 110.8 kJ/mol and 140.0 and 105.8 °C, respectively (Ar^1^ = *m*-Cl-C_6_H_4_ for both phosphonium salts). The *E*_a_ and *T*_1/2_^1h^ for the phthalimide derivative with a phenyl group in the α-position were 115.6 kJ/mol and 63.3 °C. These results suggest that the *E*_a_ and *T*_1/2_^1h^ corresponding to the α-formation of imidocarbenium cations decrease as the cation stability increases. The α-imidocarbenium cations derived from phthalimide should be more stable than the corresponding succinimide derivatives owing to the additional resonance stabilization provided by the phenylene group. The presence of a phenyl group rather than a methyl group at the α-position (compound **6e**) causes the benzyl-type resonance stabilization of the α-imidocarbenium cation (the significant reduction of *T*_1/2_^1h^; 105.8 °C (Me) vs 63.3 °C (Ph)).

**Figure 2:**
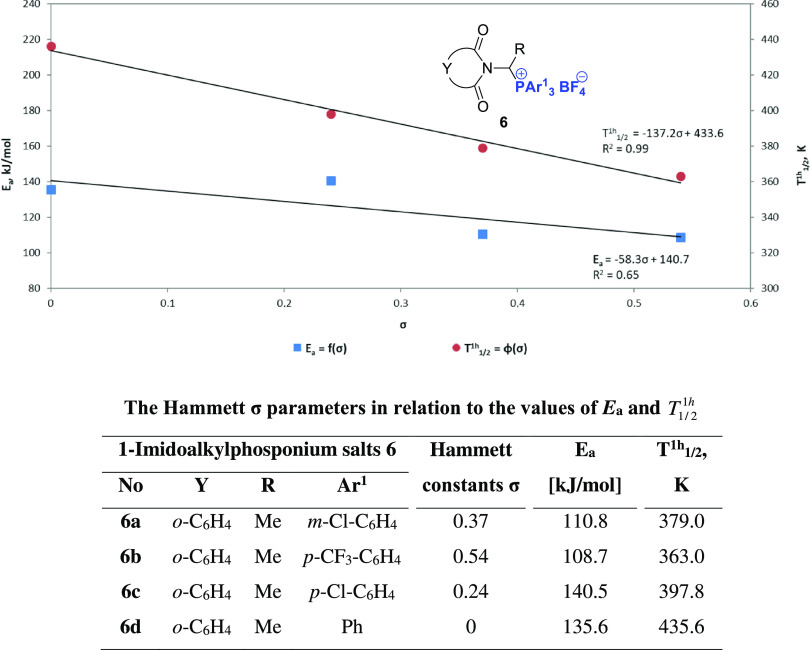
Relationships between *E*_a_ and *T*_1/2_^1h^ and the Hammett σ parameters for the generation of 1-imidocarbenium cations **7** from 1-imidoalkylphosponium salts **6**.

We also found that the strength of the C_α_–P^+^ bond can be reduced and thus facilitate the generation of the imidocarbenium cation **7**, by introducing electron-withdrawing substituents to the aryl group of the initial 1-imidoalkyltriarylphosphonium salt. The *E*_a_ and *T*_1/2_^1h^ of the 1-phthalimidoethylphosphonium salts **6** derived from triphenylphosphine (Ar^1^ = Ph), tris(*p*-chlorophenyl)phosphine (Ar^1^ = *p*-Cl-C_6_H_4_), tris(*m*-chlorophenyl)phosphine (Ar^1^ = *m*-Cl-C_6_H_4_), and tris(*p*-trifluormethylphenyl)phosphine (Ar^1^ = *p*-CF_3_-C_6_H_4_) are equal to 135.6, 140.5, 110.8, and 108.7 kJ/mol, and 162.4, 124.6, 105.8, and 89.8 °C, respectively. These values are consistent with the Hammett σ parameters describing the electron-withdrawing efficiency of the corresponding substituents, i.e., 0, 0.24, 0.37, and 0.54, respectively (Figure 2).^17^

The coefficient of determination for *E*_a_ = *f*(σ) is rather low (*R*^2^ = 0.65) in contrast to that for *T*_1/2_^1h^ = ϕ(σ). The linearity of the latter function is much better (*R*^2^ = 0.99), which emphasizes the practical meaning of the *T*_1/2_^1h^ (both parameters of the Arrhenius equation are included for its determination—see the eq 3, better correlations, practical utility—direct relation to the reaction temperature).

Thus, highly reactive imidocarbenium cations **7** can effectively be generated at a relatively low temperature (<110 °C) from 1-imidoalkyltriarylphosphonium salts derived from tris(*m*-chlorophenyl)phosphine **6a** or tris(*p*-trifluormethylphenyl)phosphine **6b**.

Finally, we defined the limit of reactivity for 1-imidoalkylphosphonium salts derived from tris(*p*-trifluormethylphenyl)phosphine **6b** in the Friedel–Crafts-type (Tscherniac–Einhorn-type) imidoalkylation of aromatic hydrocarbons with different levels of activation (Table 2). We recently demonstrated that 1-imidoalkylphosphonium salts **6b** reacted easily with anisole or 1,3-dimethoxybenzene to afford the expected products with good to excellent yields (for anisole: 90 °C/2 h/91%; for 1,3-dimethoxybenzene: 80 °C/3 h/82%).^13^ However, reactions of 1-imidoalkylphosphonium salt **6b** with toluene at 130 °C afforded the expected product in a 23% yield along with the corresponding enimide **9** (54%) after 2 h. In the reaction between **6b** and benzene (150 °C, 2 h), only trace amounts of the imidalkylation product were detected (<5%), and enimide **9** was the main product (61%). The reaction of imidoalkylphosphonium salt **6b** with chlorobenzene (150 °C, 2 h) gave the corresponding enimide **9** as the only reaction product (53%).

**Table 2:**
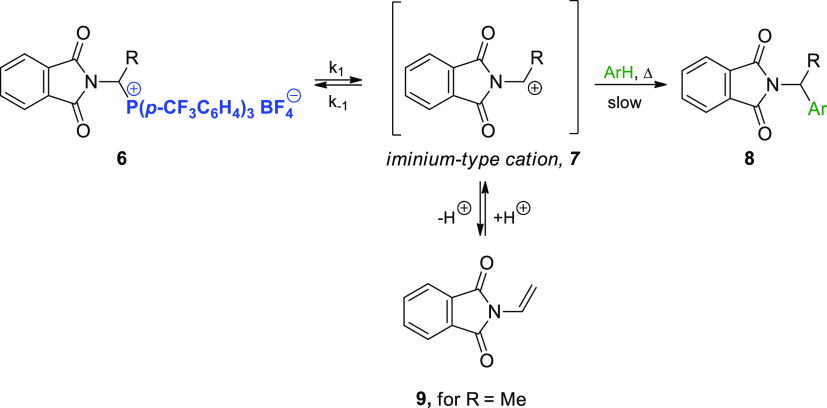
Conditions and Yields of Reactions between 1-Imidoalkyltriarylphosphonium Salts **6** and Less-Activated, Inactivated, or Deactivated Arenes

	**6**								
entry	Nr	R	ArH	solvent/acid	T, °C	time, h	8	yield, %	o:*p*^a^
1	**6b**	Me	toluene		130	2	**8ba + 8bb**	23^b^	1.0:2.6
2	**6b**	Me		-/TfOH (1 eq)	110	2		52	1.0:2.9
3	**6b**	Me		-/TfOH (1 eq)	120	1		48	1.0:2.9
4	**6b**	Me		C_6_H_5_NO_2_/TfOH (1eq)	110	2		48	1.0:2.8
5	**6g**	Ph		C_6_H_5_NO_2_/-	130	2	**8ga + 8gb**	51	1.0:2.3
6	**6b**	Me	benzene		150	2	**8bc**	traces^c^	
7	**6b**	Me		-/TfOH (1 eq)	150	2		25	
8	**6b**	Me		-/TfOH (2 eq)	130	6		32	
9	**6b**	Me		-/TfOH (2 eq)	150	2		34	
10	**6b**	Me	chlorobenzene		150	2	**8bd + 8be**	no product^d^	
11	**6b**	Me		-/TfOH (1 eq)	130	2		no product	
12	**6b**	Me		-/TfOH (1 eq)	150	2		traces	
13	**6b**	Me		-/TfOH (2 eq)	130	6		<10^e^	1.0:3.4
14	**6b**	Me		-/TfOH (2 eq)	150	2		<10^e^	1.0:3.0
15	**6b**	Me	nitrobenzene		190	2		no product^f^	
16	**6b**	Me		-/TfOH (2 eq)	190	2		no product^f^	

a Molar ratio of *ortho-* to *para-* isomers.

b *N*-Vinylphthalimide **9** was also obtained in 54% yield as a side product.

c *N*-Vinylphthalimide **9** was detected (61%).

d Only *N*-vinylphthalimide **9** was detected (53%).

e Attempts to isolate a pure analytical sample failed.

f Decomposition was observed.

The comparison of the above results with the measured 1-imidocarbenium cation formation rates (Table 1 and Figure 1) suggests that already in the case of toluene, and especially benzene and chlorobenzene (Table 2, entries 1, 6, and 10), the reactivities of these aromatic hydrocarbons are too low to effectively trap the 1-imidocarbenium cation; the rate of cation generation is no more the reaction-determining step. As a consequence, the majority of the generated 1-imidocarbenium cation is converted to the relatively stable enimide **9**, following proton abstraction from the adjacent carbon.

To suppress enimide formation, the aforementioned reactions were evaluated again in the presence of trifluoromethanesulfonic acid (TfOH). However, the role of TfOH is probably more complex and may also rely on additional activation by protonation of compounds **6** and **7** in a similar way, as described by Olah for *N*-hydroxymethylphthalimide.^18^

Under these conditions, we obtained imidoalkylation products of toluene, benzene, and chlorobenzene in yields of 48–52, 25–34, and ∼10%, respectively, whereas attempts of imidoalkylation of nitrobenzene failed.

In the case of benzene and chlorobenzene, it is preferable to use more TfOH (2 equiv.). With each reaction, it was necessary to increase the temperature significantly relative to the previously determined optimal temperature to generate iminium-type cations **7** (Table 2 vs Figure 1).

For cations **7** that are unable to convert into enimides (Table 2, entry 5, R = Ph), addition of acid is not necessary; however, the reactions with less-activated arenes (e.g., toluene) still require high temperatures (much higher than that required for cation generation).

These results demonstrate that the insufficient reactivity of α-amidoalkylating reagents toward aromatic hydrocarbons with low nucleophilicity can be overcome to a certain extent by introducing 1-imidoalkylphosphonium salts **6** derived from triarylphosphines with electron-withdrawing substituents (e.g., P(*p*-CF_3_-C_6_H_4_)_3_), which easily generate highly electrophilic 1-imidocarbenium cations **7**. However, even reagents with such high reactivity were not sufficiently active to induce reactions with deactivated aromatic systems, such as chlorobenzene or nitrobenzene.

## Conclusions

This work focused on the generation of iminium-type cations from phosphonium precursors. We showed that modifications within phosphonium groups (e.g., introducing electron-withdrawing substituents) affected the strength of the C_α_–P^+^ bond. Next, we qualitatively and quantitatively described the phenomenon whereby this bond is weakened. This can be of great importance for understanding and also improving other reactions in which the C–P^+^ bond is broken or formed (not only for α-amidoalkylation reaction).

The varying bond strengths manifest in increasing reactivity of imidoalkyltriarylphosphonium salts in the series: Ph < *p*-Cl-C_6_H_4_ < *m*-Cl-C_6_H_4_< *p*-CF_3_-C_6_H_4_. In addition, we demonstrated that for the reaction between phosphonium salts and activated aromatic compounds (e.g., anisole, 1,3-dimethoxybenzene, 1,3,5-trimethoxybenzene), the generation of iminium-type cations following the cleavage of the C_α_–P^+^ bond followed first-order kinetics and was the rate-limiting step. We determined the kinetic parameters for this type of transformation, including rate constants, Arrhenius equation parameters, and *T*_1/2_^1h^, a very useful parameter from the practical point of view. Next, we correlated them with the Hammett σ coefficients.

Finally, we confirmed that dicarbonyl protection (*N*-protecting groups, imidoalkyltriarylphosphonium salts) increased the electrophilicity of the resulting iminium-type cation and enabled the alkylation of less-activated or even inactivated aromatic systems (e.g., toluene, benzene). However, attempts to carry out the reaction with deactivated systems, such as chlorobenzene or nitrobenzene, failed.

## Experimental Section

### General Methods

^1^H- and ^13^C-NMR spectra were recorded at operating frequencies of 400 and 100 MHz, respectively, using TMS (tetramethylsilane) as the internal resonance shift standard. ^31^P-NMR spectra were recorded at operating frequencies of 161.9 MHz with respect to H_3_PO_4_ as zero ppm. All chemical shifts (δ) are reported in ppm and coupling constants (*J*) in Hz. Infrared (IR) spectra were measured on a Fourier transform (FT)-IR spectrophotometer (attenuated total reflectance–ATR method). Solvents (ACS grade) were stored over molecular sieves before use. All other commercially available reagents were used as received, without further purification or modifications.

### Substrate Synthesis

The 1-imidoalkylphosphonium salts **6a**–**6g** were synthesized according to our group’s previously described procedure.^13^

#### Generation of Imidocarbenium Cations—Kinetic Measurements

To a solution of 1-(*N*-imido)alkyltriarylphosphonium salts **6** (0.08 mmol) in nitrobenzene (400 μL) in a glass vial sealed with a screw-cap, arenes (anisole, 1,3-dimethoxybenzene, 1,3,5-trimethoxybenzene; 1.19 mmol) or a mixture thereof (in a molar ratio of 1:1) were added. Dimethyldiphenylsilane (5 mg) was used as the internal standard. The reaction mixture was vigorously stirred and heated under the conditions given in Table 1. At appropriate time intervals, an aliquot of the reaction mixture (75 μL) was removed and dissolved in 0.6 cm^3^ of CDCl_3_. Changes in the concentrations of the substrate and/or products were monitored by ^1^H-NMR and confirmed by ^31^P-NMR spectroscopy.

The spectroscopic characterization of the substrates and products is well established and was described in our group’s previous work.^13^

#### Imidoalkylation of Aromatic Hydrocarbons—General Procedure

To 1-(*N*-imido)alkyltriarylphosphonium salt **6** (0.1 mmol) in a glass vial sealed with a screw-cap, an aromatic compound (2 cm^3^) was added. In some experiments, an additional solvent (1/1; v/v; 1 cm^3^ of aromatic compounds/1 cm^3^ of cosolvent; Table 2) was introduced to improve the solubility of the phosphonium salts **6**. To suppress the side reaction producing *N*-vinylphthalimide from **6b** in selected reactions with toluene, benzene, chlorobenzene, or nitrobenzene, TfOH (1 or 2 eq; 0.1 or 0.2 mmol; 8.9 or 17.7 μL; Table 2) was added to the reaction mixture. Next, it was vigorously stirred and heated under the conditions given in Table 2. Volatile components were evaporated under reduced pressure, and the product was isolated by column chromatography with hexane (50 cm^3^) and then toluene/ethyl acetate (10:1, v/v).

The complete spectroscopic characterization, as well as the purification and separation methods for compounds **8ba**, **8bb**, **8ga**, and **8gb** are provided in our group’s previous report.^13^

##### *N*-[1-(4-Methylphenyl)ethyl]phthalimide (**8ba**) and *N*-[1-(2-methylphenyl)ethyl]-phthalimide (**8bb**)^13,19,20^

A mixture of two isomers was obtained (13.8 mg, 52% yield).

Major *p*-isomer (**8ba**): ^1^H-NMR (400 MHz, CDCl_3_) δ 7.83–7.70^a^ (m, 2H), 7.72–7.64^a^ (m, 2H), 7.43–7.36 (m, 2H), 7.17–7.10 (m, 2H), 5.54 (q, *J* = 7.3 Hz, 1H), 2.31 (s, 3H), 1.91 (d, *J* = 7.3 Hz, 3H) ppm; ^13^C-NMR (100 MHz, CDCl3) δ 168.1, 137.3, 137.3, 133.8, 132.0, 129.1, 127.4, 123.1, 49.4, 21.0, 17.5 ppm.

Minor *o*-isomer (**8bb**): ^1^H-NMR (400 MHz, CDCl_3_) δ 7.83–7.70^a^ (m, 2H), 7.72–7.64^a^ (m, 2H), 7.32–7.04^a^ (m, 4H), 5.72 (q, *J* = 7.9 Hz, 1H), 2.37 (s, 3H), 1.88 (d, *J* = 7.3 Hz, 3H) ppm. ^a^Overlapping signals of two isomers.

##### *N*-[1-(4-Methylphenyl)-1-phenylmethyl]phthalimide (**8ga**) and *N*-[1-(2-methylphenyl)-1-phenylmethyl]phthalimide (**8gb**)^13^

A mixture of two isomers was obtained (16.7 mg, 51% yield).

Major *p*-isomer (**8ga**): ^1^H-NMR (400 MHz, CDCl_3_) δ 7.86–7.80^a^ (m, 2H), 7.74–7.67^a^ (m, 2H), 7.41–7.26^a^ (m, 7H), 7.16–7.09 (m, 2H), 6.68 (s, 1H), 2.33 (s, 3H) ppm; ^13^C-NMR (100 MHz, CDCl3) δ 167.9, 138.4, 137.4, 135.2, 134.0, 131.9, 129.0, 128.7, 128.6, 128.3, 127.6, 123.4, 57.6, 21.1 ppm.

Minor *o*-isomer (**8gb**): ^1^H-NMR (400 MHz, CDCl_3_) δ 7.86–7.80^a^ (m, 2H), 7.74–7.67^a^ (m, 2H), 7.41–7.26^a^ (m, 7H), 7.23–7.16 (m, 2H), 6.83 (s, 1H), 2.29 (s, 3H) ppm. ^a^Overlapping signals of two isomers.

##### *N*-(1-Phenylethyl)phthalimide (**8bc**)^20^

Colorless oil (8.5 mg, 34%). ^1^H-NMR (400 MHz, CDCl_3_) δ 7.87–7.74 (m, 2H), 7.73–7.63 (m, 2H), 7.54–7.45 (m, 2H), 7.37–7.28 (m, 2H), 7.27–7.21 (m, 1H), 5.57 (q, *J* = 7.3 Hz, 1H), 1.93 (d, *J* = 7.3 Hz, 3H) ppm; ^13^C-NMR (100 MHz, CDCl_3_) δ 168.1, 140.3, 133.9, 132.0, 128.5, 127.7, 127.4, 123.2, 49.6, 17.5 ppm. IR (ATR) 3033, 1773, 1705, 1611, 1467, 1385, 1354, 1322, 1133, 1027, 718 cm^–1^.

##### N-Vinylphthalimide (**9**)^21^

White wax (9.4 mg, 54%). ^1^H-NMR (400 MHz, CDCl_3_) δ 7.90–7.84 (m, 2H), 7.76–7.72 (m, 2H), 6.88 (dd, *J*_1_ = 16.4 Hz, *J*_2_ = 9.6 Hz, 1H), 6.04 (d, *J* = 16.4 Hz, 1H), 5.05 (d, *J* = 9.6 Hz, 1H) ppm; ^13^C-NMR (100 MHz, CDCl_3_) δ 166.5, 134.5, 133.9, 123.6, 123.2, 104.5 ppm; IR (ATR) 1782, 1717, 1637, 1468, 1379, 1323, 1305, 1021 cm^–1^.

## Supporting Information Available

Kinetic measurements/data for phosphonium salts**6**; changes in the concentration of phosphonium salt **6a** observed in ^1^H-NMR spectra during the reaction between phosphonium salt **6a** and mixture of anisole and 1,3-dimethoxybenzene at 100 °C (Figure S1); changes in the concentration of phosphonium salt **6a** observed in ^1^H-NMR spectra during the reaction between phosphonium salt **6a** and 1,3-dimethoxybenzene at 110 °C (Figure S2); changes in the concentration of phosphonium salt **6b** observed in ^1^H-NMR spectra during the reaction between phosphonium salt **6b** and 1,3-dimethoxybenzene at 92 °C (Figure S3); changes in the concentration of phosphonium salt 6e observed in ^31^P-NMR spectra during the reaction between phosphonium salt **6e** and 1,3-dimethoxybenzene at 60 °C (Figure S4) (PDF)
