# Supplementary material for: Generation and Reactivity of 1-Imidocarbenium Cations in the Friedel–Crafts-type Reaction
Source: ACS Omega. 2022 Aug 15;7(34):30486–94. doi: 10.1021/acsomega.2c03930 (PMC9434785; doi:10.1021/acsomega.2c03930)
Supplement: Supplementary file 1 — ao2c03930_si_001.pdf [file ao2c03930_si_001.pdf]

Supporting Information

Generation and reactivity of 1-imidocarbenium cations in the Friedel-Crafts-type reaction

Jakub Adamek,[1,2,] Roman Mazurkiewicz,[1] Anna Węgrzyk-Schlieter[1]*

[1]Department of Organic Chemistry, Bioorganic Chemistry and Biotechnology, Silesian University of Technology, Bolesława Krzywoustego 4, 44-100 Gliwice, Poland

[2]Biotechnology Center, Silesian University of Technology, Bolesława Krzywoustego 8, 44-100 Gliwice, Poland

*Corresponding author: e-mail: jakub.adamek@polsl.pl

Experimental and analytical data

Table of contents

1. Apparatus for the kinetic measurements………..……………………………………………….…...……………....S2
2. Kinetic measurements/data…..…………………………………………………………………………..S3-S18

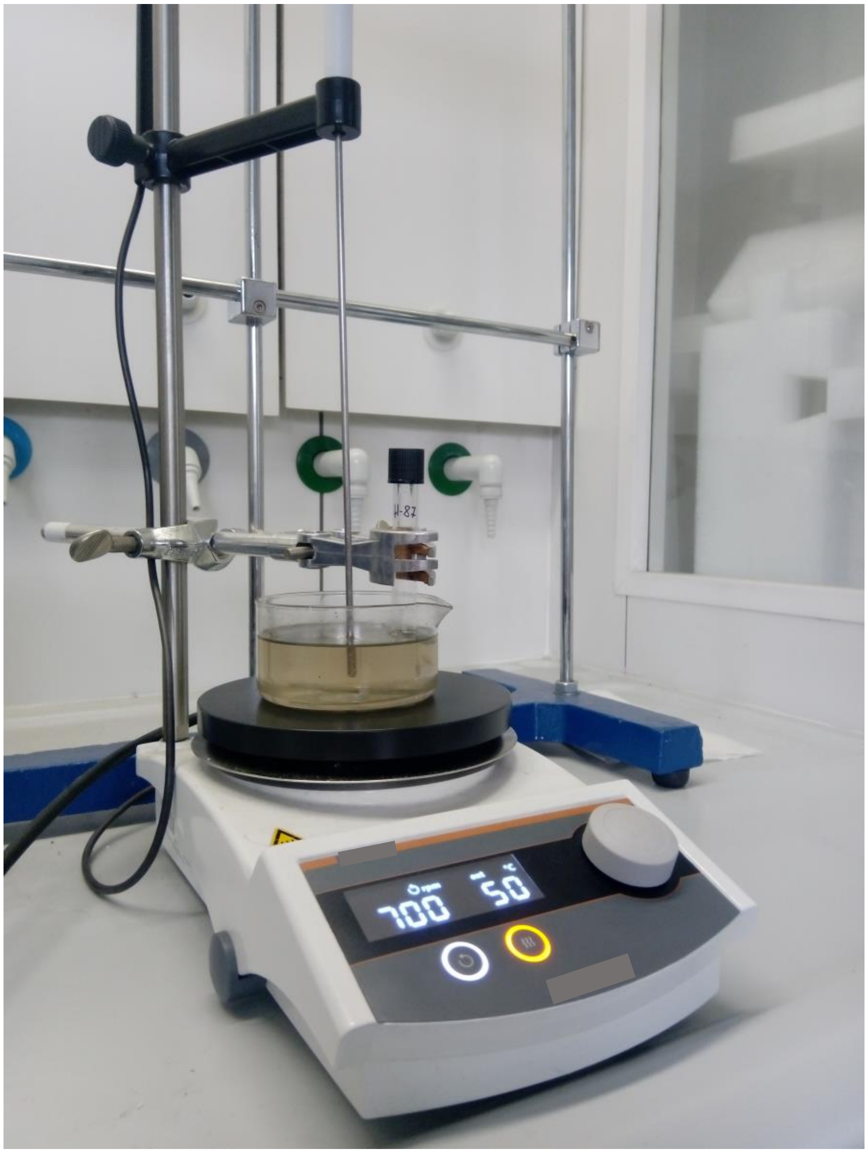

Photo S1. Apparatus for the kinetic measurements (personal photo made by authors).

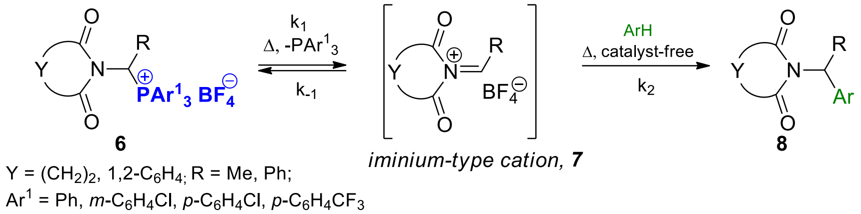

Table S1. Rate constants for the reaction of 1-imidoalkylphosphonium salts **6** with aromatic compounds.

Entry	1-Imidoalkylphosponium salts[a] No	Y	R	Ar^1	ArH[b]	Cosolvent[c]	Temp. [°C]	$k_1 \cdot 10^4$ [s^{-1}]
1	**6a**	*o*-C_6H_4	Me	*m*-Cl-C_6H_4	C_6H_5OMe	$PhNO_2$	100	0.80
2	**6a**	*o*-C_6H_4	Me	*m*-Cl-C_6H_4	1,3-$C_6H_4(OMe)_2$	$PhNO_2$	100	1.08
3	**6a**	*o*-C_6H_4	Me	*m*-Cl-C_6H_4	1,3,5-$C_6H_3(OMe)_3$	$PhNO_2$	100	1.20
4	**6a**	*o*-C_6H_4	Me	*m*-Cl-C_6H_4	C_6H_5OMe	$PhNO_2$	120	7.50
5	**6a**	*o*-C_6H_4	Me	*m*-Cl-C_6H_4	1,3-$C_6H_4(OMe)_2$	$PhNO_2$	120	6.86
6	**6a**	*o*-C_6H_4	Me	*m*-Cl-C_6H_4	1,3,5-$C_6H_3(OMe)_3$	$PhNO_2$	120	10.3
7	**6a**	*o*-C_6H_4	Me	*m*-Cl-C_6H_4	C_6H_5OMe + 1,3-$C_6H_4(OMe)_2$[d]	$PhNO_2$	100	1.09[e]
8	**6a**	*o*-C_6H_4	Me	*m*-Cl-C_6H_4	1,3-$C_6H_4(OMe)_2$ + 1,3,5-$C_6H_3(OMe)_3$[d]	$PhNO_2$	100	1.31[f]
9	**6b**	*o*-C_6H_4	Me	*p*-CF_3-C_6H_4	1,3-$C_6H_4(OMe)_2$	$PhNO_2$	100	6.0
10	**6b**	*o*-C_6H_4	Me	*p*-CF_3-C_6H_4	1,3-$C_6H_4(OMe)_2$	C_6H_5Me	100	18.7
11	**6b**	*o*-C_6H_4	Me	*p*-CF_3-C_6H_4	1,3-$C_6H_4(OMe)_2$	None[g]	100	16.1
**Kinetic data for entries 12-15 are given on pages: S14 (6c), S15 (6d), S16 (6e), and S18 (6f)**								
12	**6c**	*o*-C_6H_4	Me	*p*-Cl-C_6H_4	1,3-$C_6H_4(OMe)_2$	$PhNO_2$	120	1.37
13	**6d**	*o*-C_6H_4	Me	Ph	1,3-$C_6H_4(OMe)_2$	$PhNO_2$	160	1.68
14	**6e**	*o*-C_6H_4	Ph	*m*-Cl-C_6H_4	1,3-$C_6H_4(OMe)_2$	$PhNO_2$	60	1.24
15	**6f**	$(CH_2)_2$	Me	*m*-Cl-C_6H_4	1,3-$C_6H_4(OMe)_2$	$PhNO_2$	140	1.81

[a]0.08 mmol; [b]1.19 mmol (129 µl of anisole, 156 µl of 1,3-dimethoxybenzene or 200 mg of 1,3,5-trimethoxybenzene); [c]400 µl; [d]Molar ratio of 1:1 (0.6 mmol:0.6 mmol); [e]No traces of α-imidoalkylation product of anisole were detected; [f]A mixture of α-imidoalkylation products of 1,3-$C_6H_4(OMe)_2$ and 1,3,5-$C_6H_4(OMe)_3$ in a molar ratio of 1:2; [g]Additional amount of 400 µl of 1,3-$C_6H_4(OMe)_2$ was used instead of a cosolvent.

Table S2. Entries 1-3; 100 °C

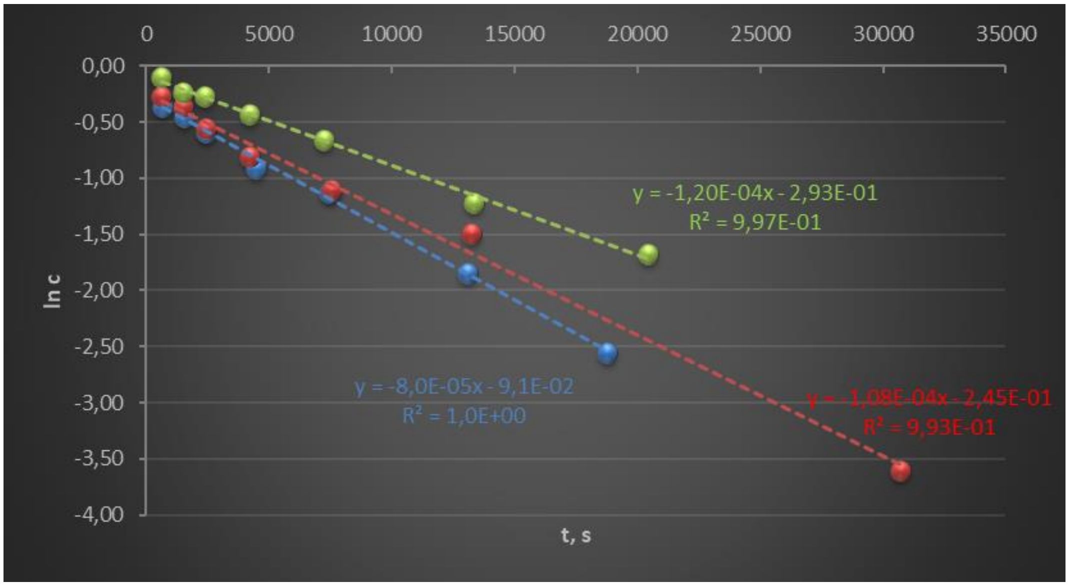

time, s	Substrate S	Standard D	ln(S/D)	regression analysis	
	integral value in ^{1}H-NMR		concentration change		
anisole					
609	45,02	49,69	-0,10	-7,96E-05	-9,07E-02
1507	39,65	50,10	-0,23	2,26E-06	2,21E-02
2400	36,99	48,78	-0,28	9,96E-01	4,04E-02
4200	31,50	48,35	-0,43	1,24E+03	5,00E+00
7233	25,08	48,60	-0,66	2,02E+00	8,16E-03
13304	15,29	51,52	-1,21		
20400	9,86	52,56	-1,67		
1,3-dimethoxybenzene					
607	38,94	50,97	-0,27	-1,08E-04	-2,45E-01
1506	32,22	46,52	-0,37	3,98E-06	5,22E-02
2407	27,21	47,41	-0,56	9,93E-01	1,04E-01
4208	21,00	47,14	-0,81	7,31E+02	5,00E+00
7513	15,26	46,09	-1,11	7,93E+00	5,43E-02
13213	10,33	45,89	-1,49		
30720	1,32	48,38	-3,60		
1,3,5-trimethoxybenzene					
660	38,67	55,50	-0,36	-1,20E-04	-2,93E-01
1530	31,63	49,81	-0,45	2,79E-06	2,60E-02
2430	27,24	49,45	-0,60	9,97E-01	4,61E-02
4470	20,24	50,09	-0,91	1,84E+03	5,00E+00
7440	16,20	49,89	-1,12	3,91E+00	1,06E-02
13080	8,24	52,24	-1,85		
18780	4,21	54,14	-2,55		

Table S3. Entries 4-6, 120 °C

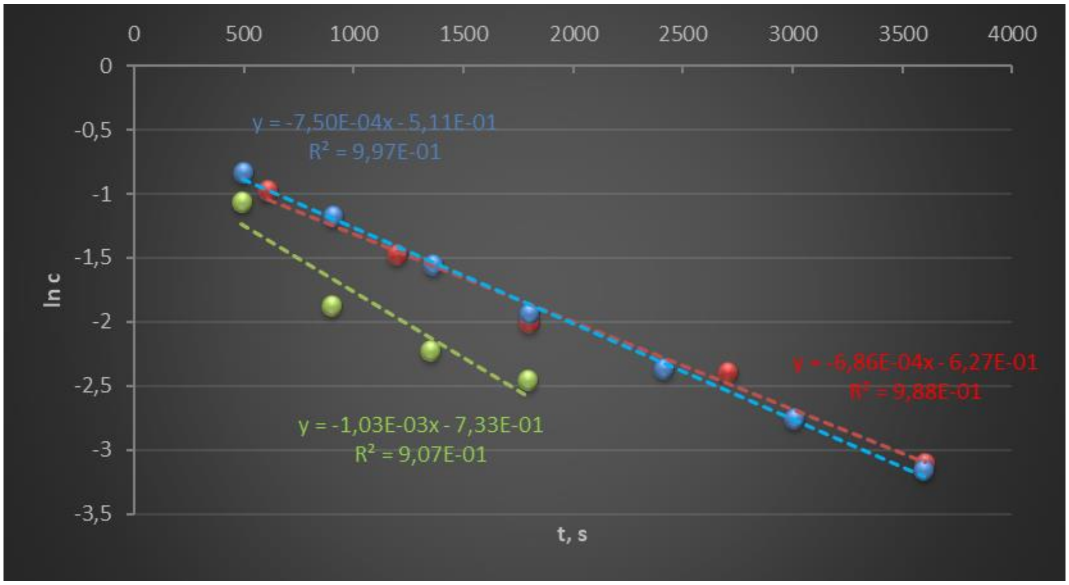

time, s	Substrate S	Standard D	ln(S/D)	regression analysis	
	integral value in ^{1}H-NMR		concentration change		
anisole					
494	25,01	57,32	-0,83	-7,50E-04	-5,11E-01
904	17,70	56,84	-1,17	1,93E-05	4,25E-02
1359	12,12	56,87	-1,55	9,97E-01	5,33E-02
1800	8,31	57,50	-1,93	1,51E+03	5,00E+00
2409	5,45	57,98	-2,36	4,29E+00	1,42E-02
3004	3,64	57,30	-2,76		
3597	2,48	58,04	-3,15		
1,3-dimethoxybenzene					
609	22,06	58,18	-0,97	-6,86E-04	-6,27E-01
1194	13,33	58,08	-1,47	4,36E-05	9,82E-02
1801	7,71	56,83	-2,00	9,88E-01	1,04E-01
2705	5,05	55,31	-2,39	2,48E+02	3,00E+00
3605	2,51	55,89	-3,10	2,68E+00	3,25E-02
1,3,5-trimethoxybenzene					
489	18,60	53,66	-1,06	-1,03E-03	-7,33E-01
898	8,11	52,83	-1,87	2,33E-04	2,87E-01
1350	5,50	50,75	-2,22	9,07E-01	2,27E-01
1790	4,44	51,56	-2,45	1,96E+01	2,00E+00

Table S4. Entries 7-8, 100 °C

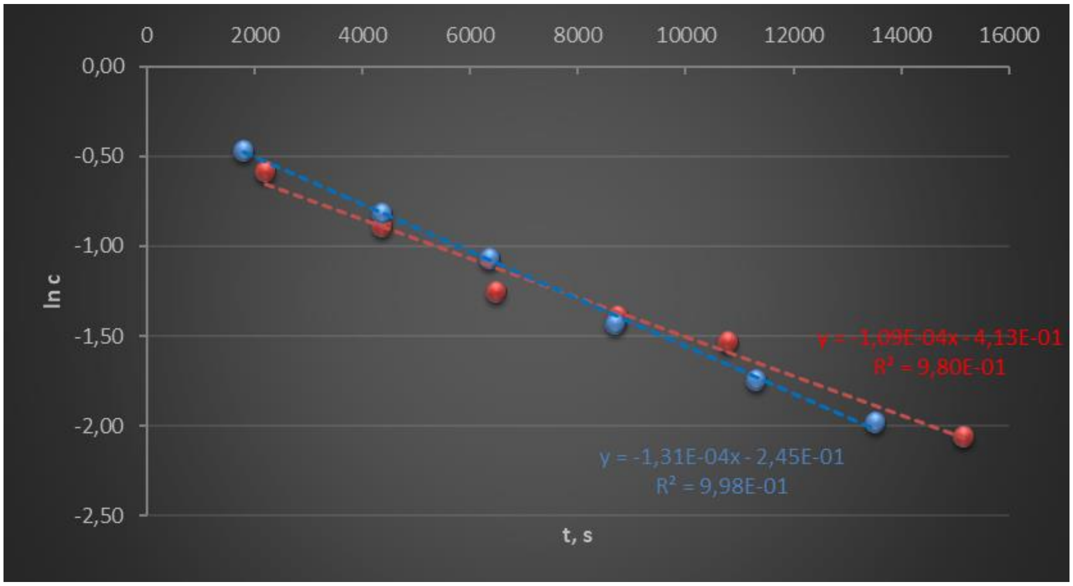

time, s	Substrate S	Standard D	ln(S/D)	regression analysis	
	integral value in ^{1}H-NMR		concentration change		
anisole/1,3-dimethoxybenzene					
2204	28,96	51,80	-0,58	-1,09E-04	-4,13E-01
4352	21,19	51,32	-0,88	7,80E-06	7,02E-02
6464	15,58	54,38	-1,25	9,80E-01	8,12E-02
8723	13,43	53,77	-1,39	1,97E+02	4,00E+00
10766	11,67	53,91	-1,53	1,30E+00	2,64E-02
15146	6,78	53,03	-2,06		
1,3-dimethoxybenzene/1,3,5-trimethoxybenzene					
1792	38,54	61,46	-0,47	-1,31E-04	-2,45E-01
4355	30,70	69,30	-0,81	3,18E-06	2,75E-02
6362	25,59	74,41	-1,07	9,98E-01	3,11E-02
8688	19,31	80,69	-1,43	1,70E+03	4,00E+00
11306	14,90	85,10	-1,74	1,64E+00	3,87E-03
13500	12,13	87,87	-1,98		

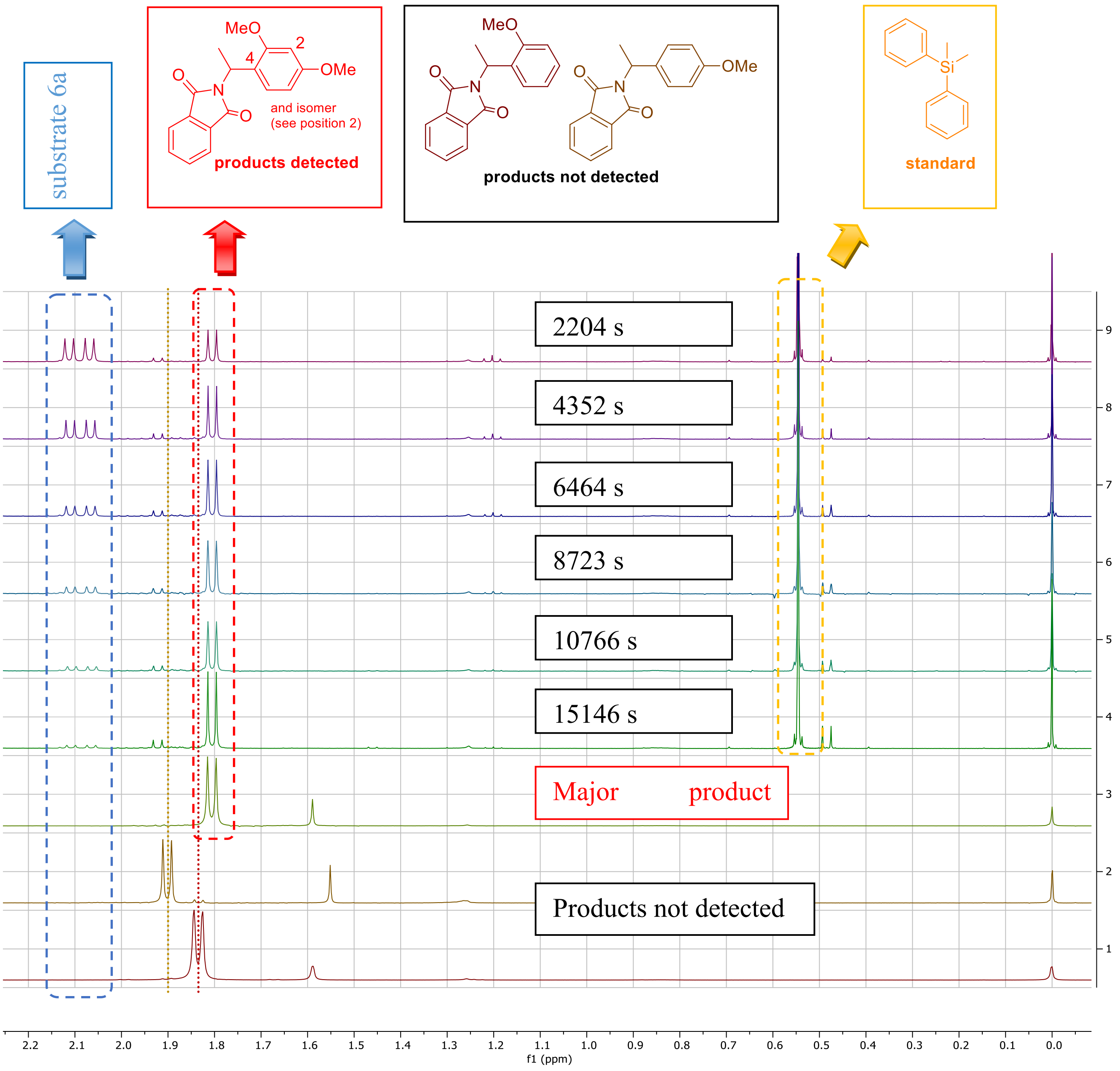

Figure S1. Changes in the concentration of phosphonium salt **6a** observed in ^{1}H-NMR spectra during the reaction between phosphonium salt **6a** and mixture of anisole and 1,3-dimethoxybenzene at 100 °C.

Table S5. Entries 9-11, 100 °C

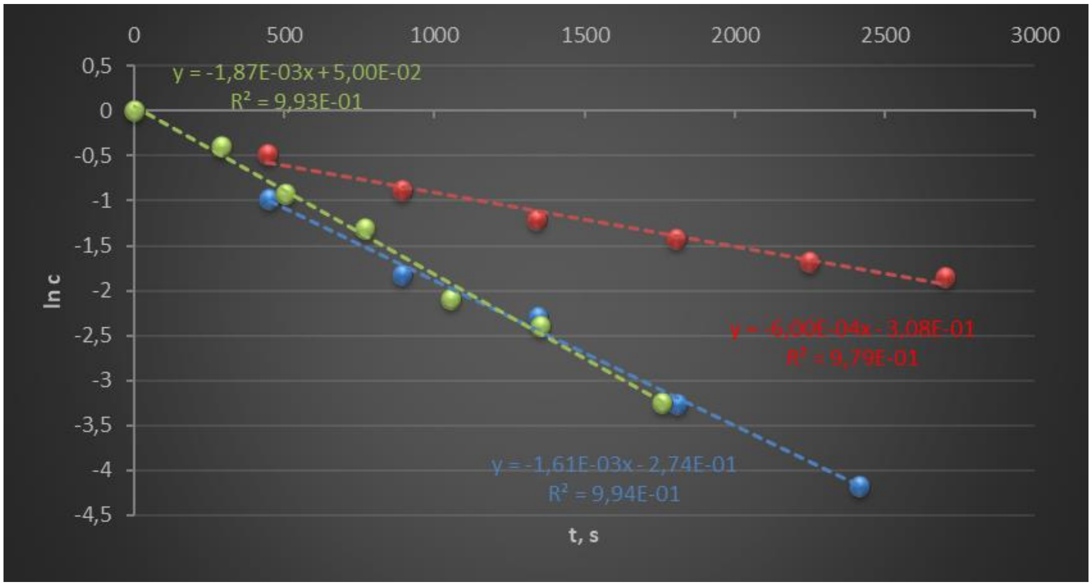

time, s	Substrate S	Standard D	ln(S/D)	regression analysis	
	integral value in ^{1}H-NMR		concentration change		
**Nitrobenzene/Temperature 100 °C (364K)**					
443	30,49	48,91	-0,47	-6,00E-04	-3,08E-01
891	19,52	46,97	-0,88	4,37E-05	7,65E-02
1341	14,11	46,88	-1,20	9,79E-01	8,26E-02
1804	11,09	46,14	-1,43	1,88E+02	4,00E+00
2250	9,91	52,9	-1,67	1,29E+00	2,73E-02
2700	7,32	46,43	-1,85		
**1,3-Dimethoxybenzene/Temperature 100 °C (364K)**					
449	19,85	53,1	-0,98	-1,61E-03	-2,74E-01
893	9,18	56,37	-1,81	7,28E-05	1,12E-01
1346	5,71	56,55	-2,29	9,94E-01	1,12E-01
1811	2,25	58,56	-3,26	4,90E+02	3,00E+00
2414	1,02	66,02	-4,17	6,14E+00	3,76E-02
time, s	Substrate S		ln(S)	regression analysis	
	integral value in ^{31}P-NMR[a]		concentration change		
**Toluene/Temperature 100 °C (364K)**					
0	1,0000		0,00	-1,87E-03	5,00E-02
289	0,6710		-0,40	6,97E-05	6,95E-02
505	0,3980		-0,92	9,93E-01	1,05E-01
768	0,2697		-1,31	7,20E+02	5,00E+00
1055	0,1228		-2,10	7,97E+00	5,53E-02
1354	0,0912		-2,39		
1759	0,0391		-3,24		

[a]Concentration changes were estimated based on ^{31}P-NMR spectra assuming that the total phosphorus content did not change; in the ^{1}H-NMR spectra, the signal from the CH_3 group of toluene made it difficult to integrate the substrate signal (also the CH_3 group).

Table S6. Arrhenius equation parameters

(measurements/data for entries 12-15/Table 1 are also included)

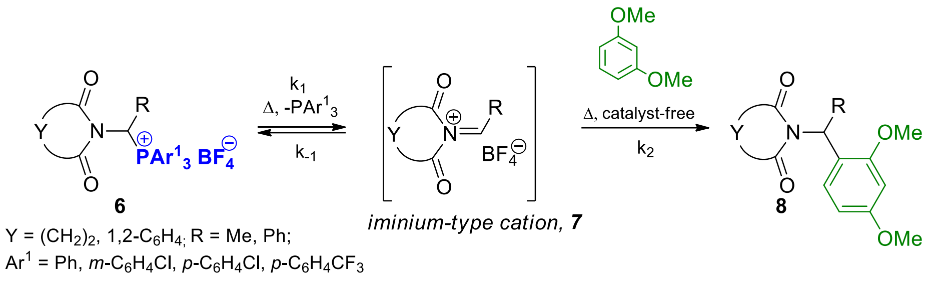

1-Imidoalkylphosponium salts 6				Arrhenius equation parameters				$T^{1h}_{1/2}$, °C	$T^{1h}_{1/2}$, K
**No.**	**Y**	**R**	**Ar¹**	**E_a, kJ/mol**	**ln A**	**A**	**R^2**		
**6a**	*o*-C_6H_4	Me	*m*-Cl-C_6H_4	110.8	26.61	$3.61 \cdot 10^{11}$	0.999	105.8	379.0
**6b**	*o*-C_6H_4	Me	*p*-CF_3-C_6H_4	108.7	27.48	$8.57 \cdot 10^{11}$	0.996	89.8	363.0
**6c**	*o*-C_6H_4	Me	*p*-Cl-C_6H_4	140.5	33.95	$5.56 \cdot 10^{14}$	0.998	124.6	397.8
**6d**	*o*-C_6H_4	Me	Ph	135.6	28.89	$3.51 \cdot 10^{12}$	0.996	162.4	435.6
**6e**	*o*-C_6H_4	Ph	*m*-Cl-C_6H_4	115.6	32.75	$1.67 \cdot 10^{14}$	0.996	63.3	336.5
**6f**	$(CH_2)_2$	Me	*m*-Cl-C_6H_4	137.0	31.32	$4.00 \cdot 10^{13}$	0.992	140.0	413.2

Table S7. Arrhenius equation parameters – kinetic measurements for **6a**

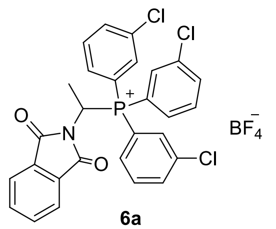

6a

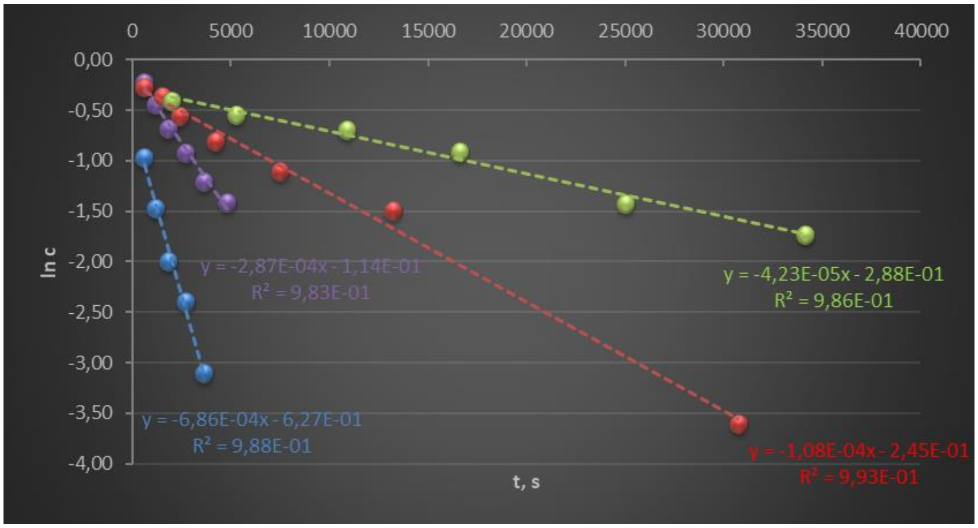

time, s	Substrate S	Standard D	ln(S/D)	regression analysis	
	integral value in ^{1}H-NMR		concentration change		
**Temperature 120 °C (393K)**					
609	22,06	58,18	-0,97	-6,86E-04	-6,27E-01
1194	13,33	58,08	-1,47	4,36E-05	9,82E-02
1801	7,71	56,83	-2,00	9,88E-01	1,04E-01
2705	5,05	55,31	-2,39	2,48E+02	3,00E+00
3605	2,51	55,89	-3,10	2,68E+00	3,25E-02
**Temperature 110 °C (383K)**					
612	38,14	47,80	-0,23	-2,87E-04	-1,14E-01
1200	30,52	47,68	-0,45	1,86E-05	5,30E-02
1804	24,41	48,36	-0,68	9,83E-01	6,54E-02
2699	18,74	47,29	-0,93	2,36E+02	4,00E+00
3610	14,27	47,63	-1,21	1,01E+00	1,71E-02
4801	11,16	45,99	-1,42		
**Temperature 100 °C (373K)**					
607	38,94	50,97	-0,27	-1,08E-04	-2,45E-01
1506	32,22	46,52	-0,37	3,98E-06	5,22E-02
2407	27,21	47,41	-0,56	9,93E-01	1,04E-01
4208	21,00	47,14	-0,81	7,31E+02	5,00E+00
7513	15,26	46,09	-1,11	7,93E+00	5,43E-02
13213	10,33	45,89	-1,49		
30720	1,32	48,38	-3,60		
**Temperature 90 °C (363K)**					
1980	37,25	55,86	-0,41	-4,23E-05	-2,88E-01
5280	30,67	52,92	-0,55	2,48E-06	4,76E-02
10860	25,30	50,47	-0,69	9,86E-01	6,77E-02
16620	20,34	50,41	-0,91	2,92E+02	4,00E+00
25020	13,14	54,51	-1,42	1,34E+00	1,83E-02
34140	9,67	54,39	-1,73		

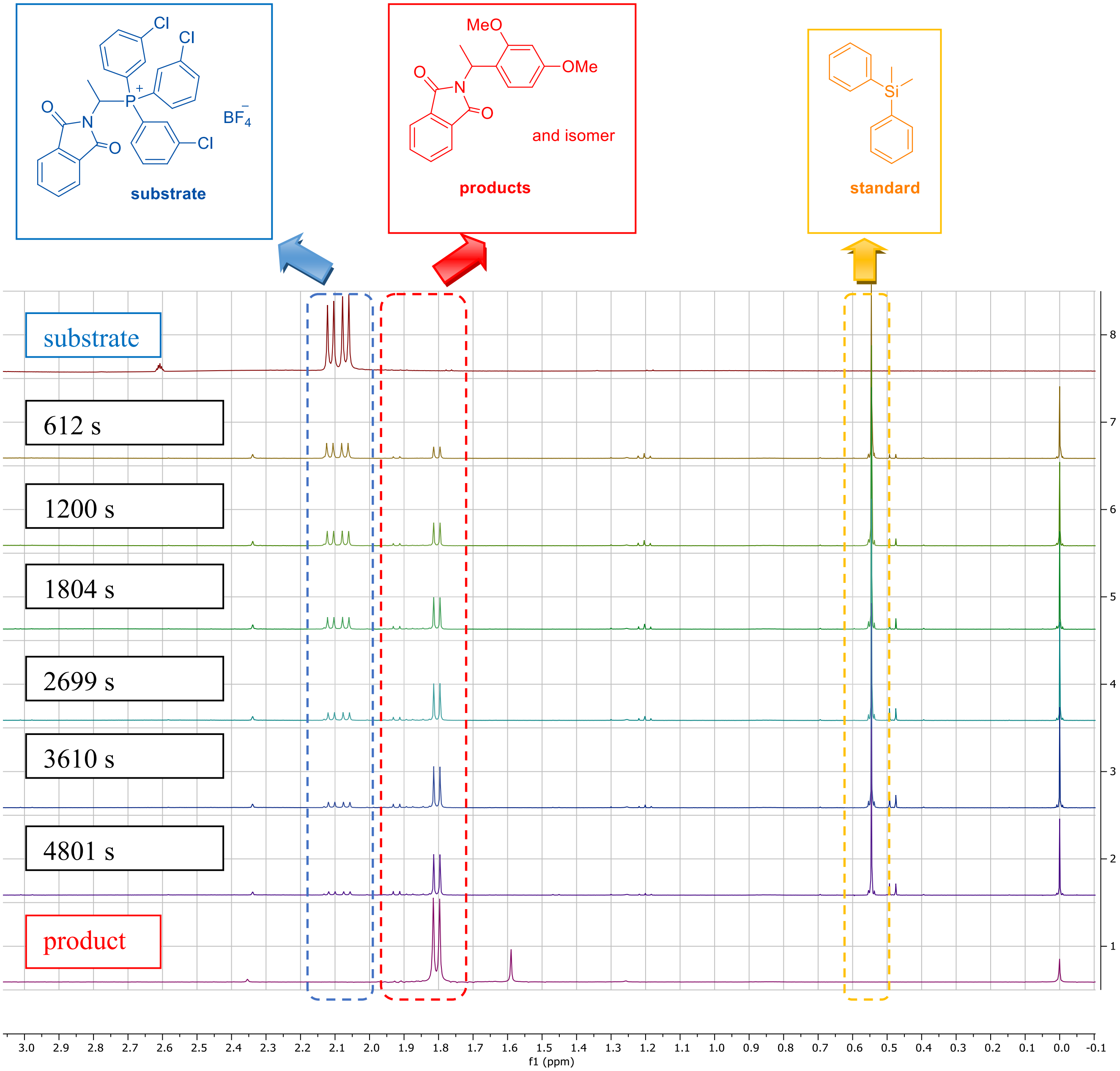

Figure S2. Changes in the concentration of phosphonium salt **6a** observed in ^{1}H-NMR spectra during the reaction between phosphonium salt **6a** and 1,3-dimethoxybenzene at 110 °C.

Table S8. Arrhenius equation parameters – kinetic measurements for **6b**

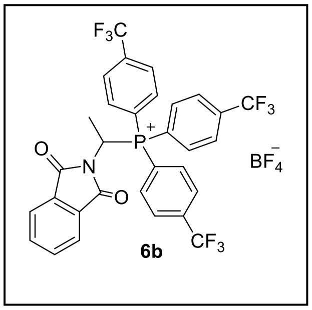

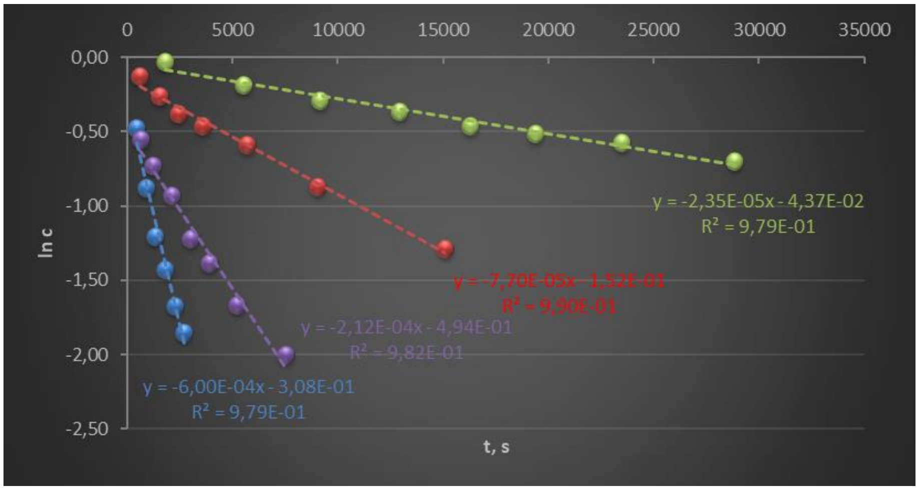

time, s	Substrate S	Standard D	ln(S/D)	regression analysis	
	integral value in ^{1}H-NMR		concentration change		
**Temperature 101 °C (374K)**					
443	30,49	48,91	-0,47	-6,00E-04	-3,08E-01
891	19,52	46,97	-0,88	4,37E-05	7,65E-02
1341	14,11	46,88	-1,20	9,79E-01	8,26E-02
1804	11,09	46,14	-1,43	1,88E+02	4,00E+00
2250	9,91	52,90	-1,67	1,29E+00	2,73E-02
2700	7,32	46,43	-1,85		
**Temperature 92 °C (365K)**					
614	31,89	55,28	-0,55	-2,12E-04	-4,94E-01
1216	26,31	54,45	-0,73	1,29E-05	5,22E-02
2093	21,03	53,25	-0,93	9,82E-01	7,64E-02
2999	15,54	52,39	-1,22	2,71E+02	5,00E+00
3917	12,92	51,39	-1,38	1,58E+00	2,92E-02
5228	9,77	51,79	-1,67		
7530	7,01	51,77	-2,00		
**Temperature 80 °C (353K)**					
599	42,76	48,70	-0,13	-7,70E-05	-1,52E-01
1549	37,88	49,28	-0,26	3,41E-06	2,46E-02
2401	34,45	50,51	-0,38	9,90E-01	4,28E-02
3600	31,25	49,52	-0,46	5,08E+02	5,00E+00
5700	27,26	49,09	-0,59	9,29E-01	9,14E-03
9036	21,13	50,59	-0,87		
15066	13,63	49,27	-1,29		
**Temperature 70 °C (343K)**					
1810	47,15	48,50	-0,028	-2,35E-05	-4,37E-02
5500	40,88	49,28	-0,187	1,42E-06	2,41E-02
9126	37,16	49,51	-0,287	9,79E-01	3,43E-02
12901	33,89	48,77	-0,364	2,75E+02	6,00E+00
16256	31,80	50,42	-0,461	3,24E-01	7,06E-03
19350	29,46	49,04	-0,510		
23457	27,57	48,92	-0,573		
28830	24,47	49,16	-0,698		

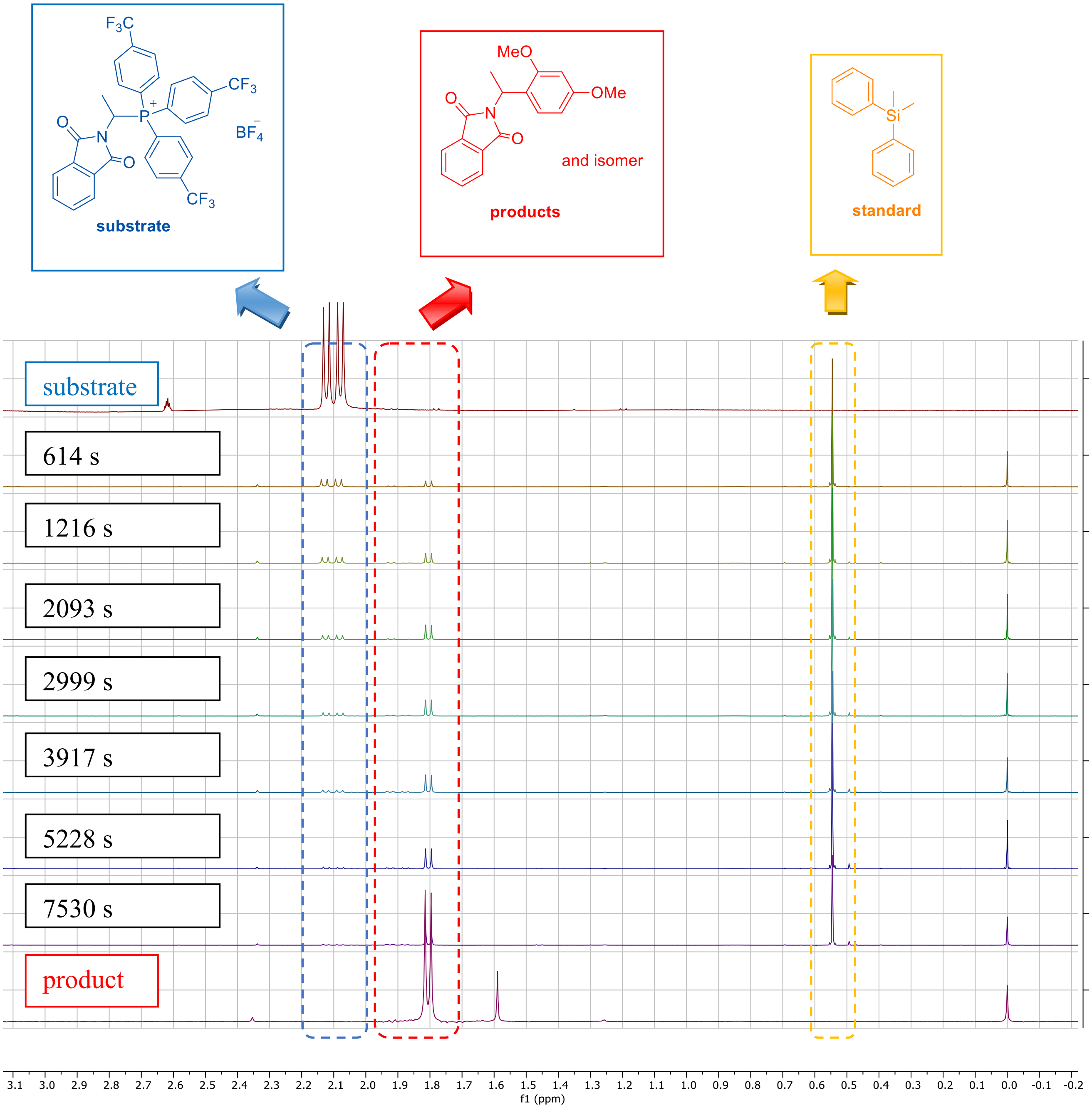

Figure S3. Changes in the concentration of phosphonium salt **6b** observed in ^{1}H-NMR spectra during the reaction between phosphonium salt **6b** and 1,3-dimethoxybenzene at 92 °C.

Table S9. Arrhenius equation parameters – kinetic measurements for **6c**

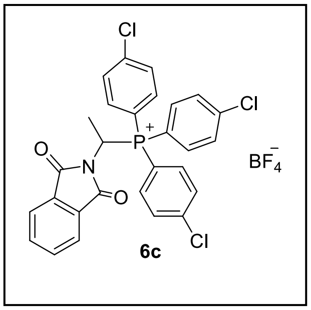

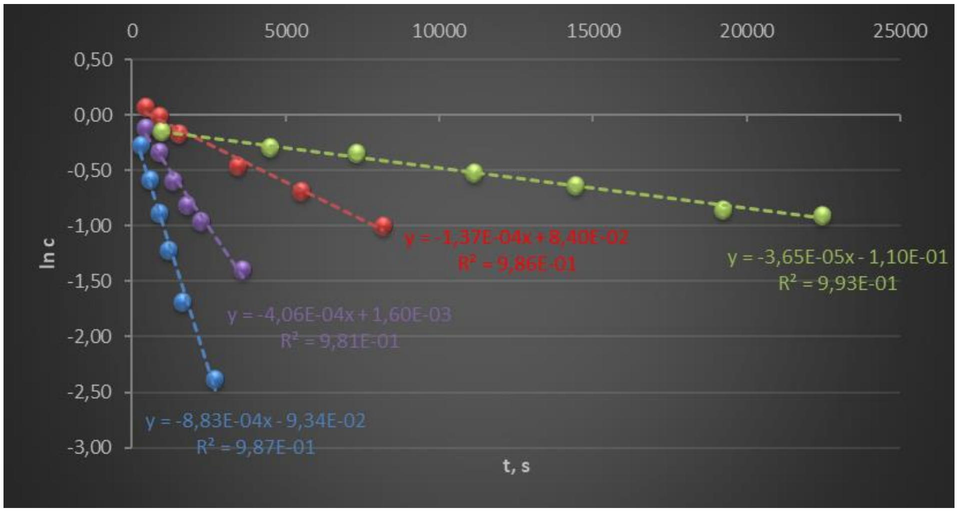

time, s	Substrate S	Standard D	ln(S/D)	regression analysis	
	integral value in ^{1}H-NMR		concentration change		
**Temperature 140 °C (413K)**					
281	43,30	56,70	-0,27	-8,83E-04	-9,34E-02
589	35,89	64,11	-0,58	5,03E-05	7,31E-02
894	29,24	70,76	-0,88	9,87E-01	9,76E-02
1192	22,93	77,07	-1,21	3,08E+02	4,00E+00
1646	15,70	84,30	-1,68	2,93E+00	3,81E-02
2700	8,47	91,53	-2,38		
**Temperature 130 °C (403K)**					
455	43,11	48,36	-0,11	-4,06E-04	1,60E-03
893	35,37	49,21	-0,33	2,82E-05	5,65E-02
1351	28,19	50,95	-0,59	9,81E-01	7,05E-02
1797	23,22	52,16	-0,81	2,07E+02	4,00E+00
2250	20,00	51,74	-0,95	1,03E+00	1,99E-02
3597	12,93	52,14	-1,39		
**Temperature 120 °C (393K)**					
458	49,86	46,13	0,08	-1,37E-04	8,40E-02
892	46,27	46,85	-0,01	8,21E-06	3,55E-02
1520	41,08	48,35	-0,16	9,86E-01	5,54E-02
3434	31,72	50,21	-0,46	2,81E+02	4,00E+00
5507	25,59	50,90	-0,69	8,62E-01	1,23E-02
8163	19,28	52,28	-1,00		
**Temperature 110 °C (383K)**					
965	46,32	53,68	-0,15	-3,65E-05	-1,10E-01
4487	42,71	57,29	-0,29	1,37E-06	1,85E-02
7288	41,49	58,51	-0,34	9,93E-01	2,62E-02
11126	37,44	62,56	-0,51	7,11E+02	5,00E+00
14425	34,60	65,40	-0,64	4,88E-01	3,43E-03
19214	29,96	70,04	-0,85		
22452	28,78	71,22	-0,91		

Table S10. Arrhenius equation parameters – kinetic measurements for **6d**

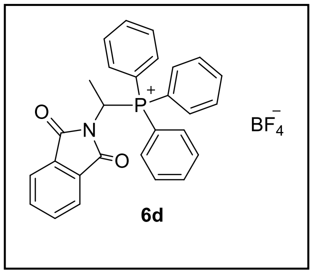

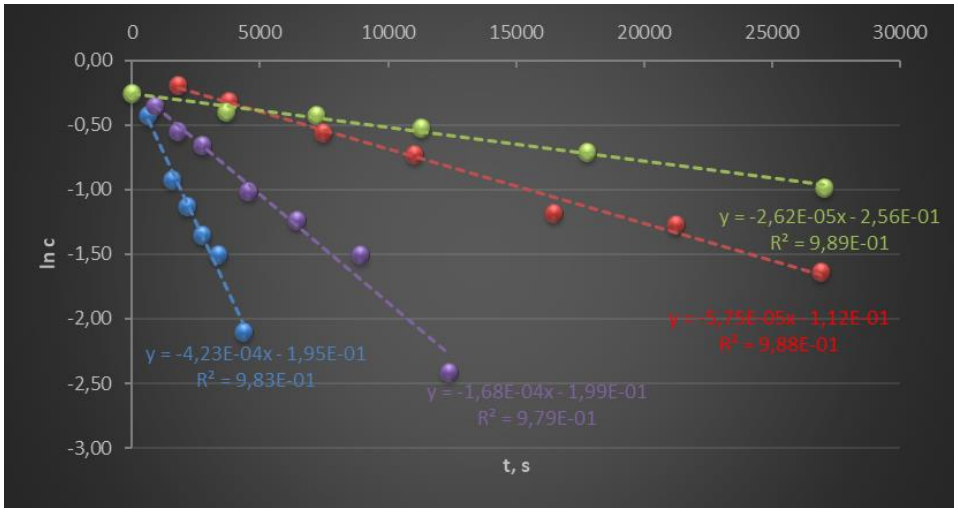

time, s	Substrate S	Standard D	ln(S/D)	regression analysis	
	integral value in ^{1}H-NMR		concentration change		
**Temperature 172 °C (445K)**					
630	33,92	51,99	-0,43	-4,23E-04	-1,95E-01
1557	20,35	51,32	-0,92	2,78E-05	7,62E-02
2135	16,61	51,08	-1,12	9,83E-01	8,21E-02
2744	13,28	51,07	-1,35	2,32E+02	4,00E+00
3380	11,01	49,34	-1,50	1,56E+00	2,70E-02
4343	6,31	51,44	-2,10		
**Temperature 160 °C (433K)**					
889	36,37	51,53	-0,35	-1,68E-04	-1,99E-01
1804	30,58	52,77	-0,55	1,09E-05	7,19E-02
2722	26,64	51,41	-0,66	9,79E-01	1,11E-01
4522	19,19	52,78	-1,01	2,38E+02	5,00E+00
6402	15,24	52,37	-1,23	2,90E+00	6,11E-02
8907	11,72	52,59	-1,50		
12370	5,01	55,67	-2,41		
**Temperature 150 °C (423K)**					
1808	41,35	50,07	-0,19	-5,75E-05	-1,12E-01
3746	35,63	48,77	-0,31	2,88E-06	4,40E-02
7443	29,6	51,54	-0,55	9,88E-01	6,55E-02
11000	24,83	51,42	-0,73	3,99E+02	5,00E+00
16440	16,74	54,69	-1,18	1,71E+00	2,15E-02
21240	14,94	53,29	-1,27		
26880	10,66	54,87	-1,64		
**Temperature 140 °C (413K)**					
0	43,7	56,3	-0,25	-2,62E-05	-2,56E-01
3667	40,21	59,79	-0,40	1,38E-06	1,98E-02
7180	39,62	60,38	-0,42	9,89E-01	3,05E-02
11298	37,2	62,8	-0,52	3,63E+02	4,00E+00
17752	32,91	67,09	-0,71	3,38E-01	3,72E-03
27025	27,25	72,75	-0,98		

Table S11. Arrhenius equation parameters – kinetic measurements for **6e**

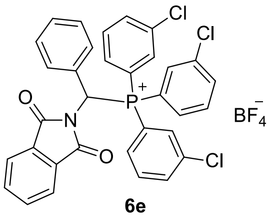

6e

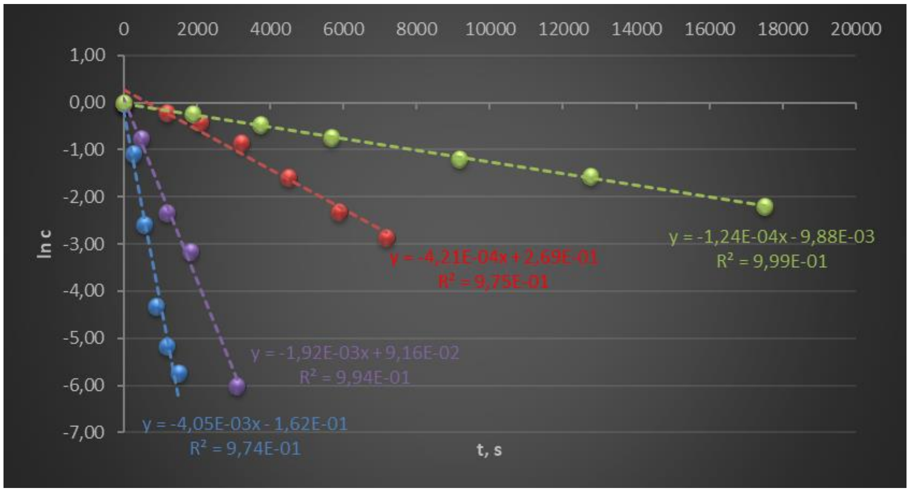

time, s	Substrate S	ln(S)	regression analysis	
	integral value in ^{31}P-NMR[a]	concentration change		
**Temperature 91 °C (364K)**				
0	1,00000	0,00	-4,05E-03	-1,62E-01
276	0,34220	-1,07	3,32E-04	2,99E-01
580	0,07480	-2,59	9,74E-01	4,18E-01
885	0,01320	-4,33	1,48E+02	4,00E+00
1190	0,00570	-5,17	2,60E+01	7,00E-01
1497	0,00330	-5,73		
**Temperature 82 °C (355K)**				
0	1,0000	0,00	-1,92E-03	9,16E-02
476	0,4641	-0,77	8,32E-05	1,42E-01
1198	0,0972	-2,33	9,94E-01	2,02E-01
1835	0,0426	-3,16	5,35E+02	3,00E+00
3104	0,0025	-6,01	2,19E+01	1,23E-01
**Temperature 70 °C (343K)**				
0	1,0000	0,00	-4,21E-04	2,69E-01
1193	0,8006	-0,22	3,04E-05	1,27E-01
2095	0,6625	-0,41	9,75E-01	1,92E-01
3207	0,4239	-0,86	1,92E+02	5,00E+00
4501	0,2035	-1,59	7,05E+00	1,83E-01
5877	0,0997	-2,31		
7178	0,0578	-2,85		
**Temperature 60 °C (333K)**				
0	1,0000	0,00	-1,24E-04	-9,88E-03
1899	0,7859	-0,24	1,56E-06	1,44E-02
3739	0,6254	-0,47	9,99E-01	2,39E-02
5690	0,4818	-0,73	6,39E+03	5,00E+00
9180	0,3040	-1,19	3,64E+00	2,85E-03
12743	0,2095	-1,56		
17520	0,1121	-2,19		

[a]Concentration changes were estimated based on ^{31}P-NMR spectra assuming that the total phosphorus content did not change; in the ^{1}H-NMR spectra there was no characteristic, easy-to-integrate signal.

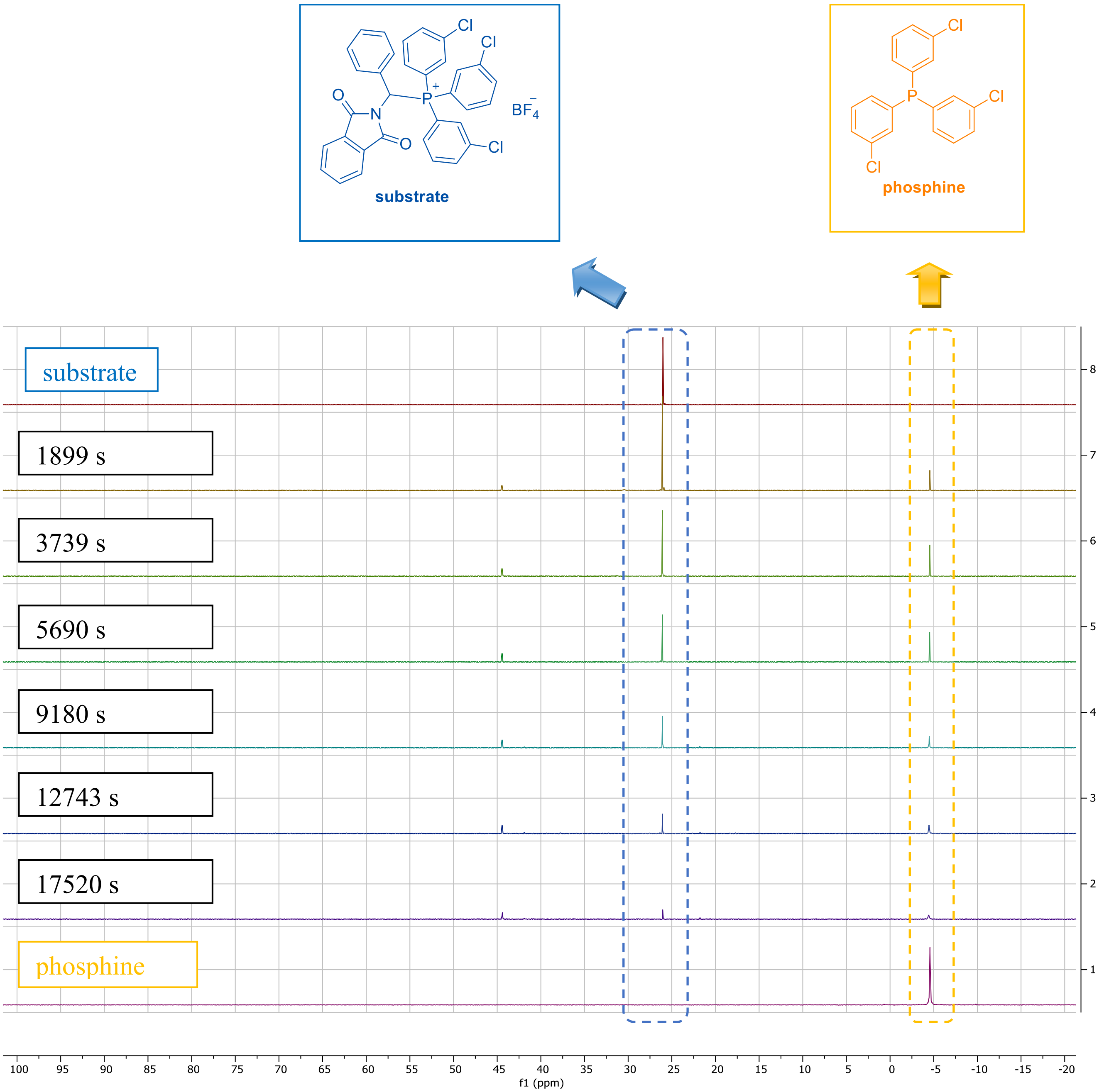

Figure S4. Changes in the concentration of phosphonium salt **6e** observed in ^{31}P-NMR spectra during the reaction between phosphonium salt **6e** and 1,3-dimethoxybenzene at 60 °C.

Table S12. Arrhenius equation parameters – kinetic measurements for **6f**

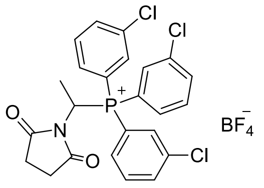

6f

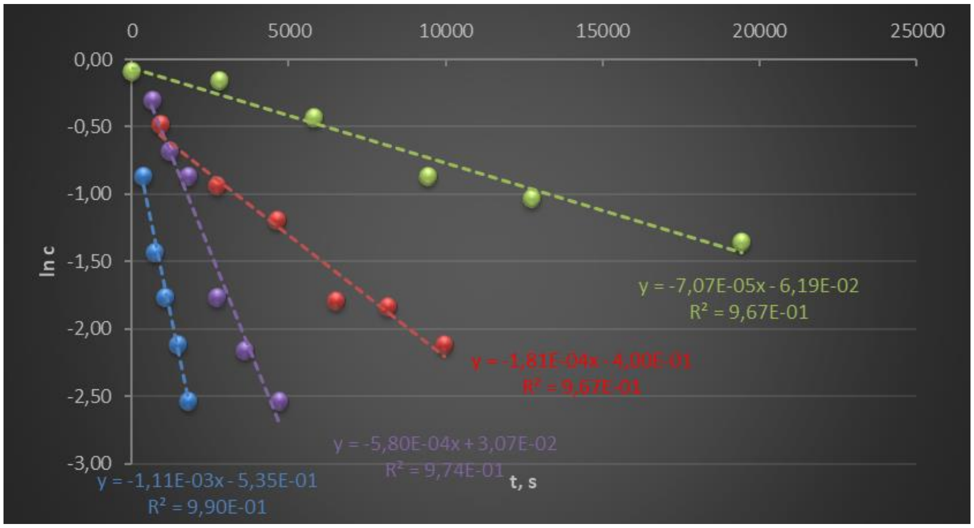

time, s	Substrate S	Standard D	ln(S/D)	regression analysis	
	integral value in 1H-NMR		concentration change		
Temperature 160 °C (433K)					
361	29,62	70,38	-0,87	-1,11E-03	-5,35E-01
731	19,31	80,69	-1,43	6,56E-05	7,88E-02
1071	14,64	85,36	-1,76	9,90E-01	7,51E-02
1473	10,8	89,2	-2,11	2,86E+02	3,00E+00
1799	7,35	92,65	-2,53	1,61E+00	1,69E-02
Temperature 150 °C (423K)					
646	42,62	57,38	-0,30	-5,80E-04	3,07E-02
1202	33,72	66,28	-0,68	4,76E-05	1,34E-01
1777	29,69	70,31	-0,86	9,74E-01	1,62E-01
2703	14,65	85,35	-1,76	1,48E+02	4,00E+00
3604	10,33	89,67	-2,16	3,91E+00	1,06E-01
4680	7,37	92,63	-2,53		
Temperature 140 °C (413K)					
902	38,16	61,83	-0,48	-1,81E-04	-4,00E-01
2698	28,23	71,77	-0,93	1,68E-05	1,05E-01
4598	23,37	76,63	-1,19	9,67E-01	1,27E-01
6476	14,37	85,63	-1,78	1,17E+02	4,00E+00
8129	13,79	86,21	-1,83	1,89E+00	6,47E-02
9949	10,77	89,23	-2,11		
Temperature 130 °C (403K)					
0	47,75	52,25	-0,09	-7,07E-05	-6,19E-02
2768	46,14	53,86	-0,15	6,48E-06	6,84E-02
5783	39,52	60,48	-0,43	9,67E-01	1,02E-01
9435	29,7	70,3	-0,86	1,19E+02	4,00E+00
12714	26,37	73,63	-1,03	1,25E+00	4,20E-02
19410	20,54	79,46	-1,35		
